# Fine mapping of type 1 diabetes regions *Idd9.1* and *Idd9.2* reveals genetic complexity

**DOI:** 10.1007/s00335-013-9466-y

**Published:** 2013-08-11

**Authors:** Emma E. Hamilton-Williams, Daniel B. Rainbow, Jocelyn Cheung, Mikkel Christensen, Paul A. Lyons, Laurence B. Peterson, Charles A. Steward, Linda A. Sherman, Linda S. Wicker

**Affiliations:** 1 Department of Immunology and Microbial Sciences, The Scripps Research Institute, 10550 North Torrey Pines Road, La Jolla, CA 92037 USA; 2 Juvenile Diabetes Research Foundation/Wellcome Trust Diabetes and Inflammation Laboratory, Department of Medical Genetics, Cambridge Institute for Medical Research, University of Cambridge, Addenbrooke’s Hospital, WT/MRC Building, Hills Road, Cambridge, CB2 0XY UK; 3 Department of Pharmacology, Merck Research Laboratories, Rahway, NJ 07065 USA; 4 The Wellcome Trust Sanger Institute, Wellcome Trust Genome Campus, Hinxton, CB10 1HH UK; 5 Present Address: University of Queensland Diamantina Institute, Translational Research Institute, Brisbane, QLD 4102 Australia; 6 Present Address: Department of Medicine and Cambridge Institute for Medical Research, University of Cambridge School of Clinical Medicine, Addenbrooke’s Hospital, Hills Road, Cambridge, CB2 0XY UK; 7 Present Address: Juvenile Diabetes Research Foundation/Wellcome Trust Diabetes and Inflammation Laboratory, Department of Medical Genetics, Cambridge Institute for Medical Research, University of Cambridge, Addenbrooke’s Hospital, Cambridge, CB2 0XY UK

## Abstract

Nonobese diabetic (NOD) mice congenic for C57BL/10 (B10)-derived genes in the *Idd9* region of chromosome 4 are highly protected from type 1 diabetes (T1D). *Idd9* has been divided into three protective subregions (*Idd9.1*, *9.2*, and *9.3*), each of which partially prevents disease. In this study we have fine-mapped the *Idd9.1* and *Idd9.2* regions, revealing further genetic complexity with at least two additional subregions contributing to protection from T1D. Using the NOD sequence from bacterial artificial chromosome clones of the *Idd9.1* and *Idd9.2* regions as well as whole-genome sequence data recently made available, sequence polymorphisms within the regions highlight a high degree of polymorphism between the NOD and B10 strains in the *Idd9* regions. Among numerous candidate genes are several with immunological importance. The *Idd9.1* region has been separated into *Idd9.1* and *Idd9.4*, with *Lck* remaining a candidate gene within *Idd9.1*. One of the *Idd9.2* regions contains the candidate genes *Masp2* (encoding mannan-binding lectin serine peptidase 2) and *Mtor* (encoding mammalian target of rapamycin). From mRNA expression analyses, we have also identified several other differentially expressed candidate genes within the *Idd9.1* and *Idd9.2* regions. These findings highlight that multiple, relatively small genetic effects combine and interact to produce significant changes in immune tolerance and diabetes onset.

**Electronic supplementary material:**

The online version of this article (doi:10.1007/s00335-013-9466-y) contains supplementary material, which is available to authorized users.

## Introduction

T1D is a T-cell-mediated autoimmune disease resulting in the destruction of the insulin-producing islet β cells of the pancreas. Nonobese diabetic (NOD) mice spontaneously develop a form of T1D controlled by more than 20 independent gene regions, each with one or more insulin-dependent diabetes (*Idd*) genes. Considerable overlap exists between disease loci in the NOD mouse and humans with shared susceptibility gene pathways, including genes encoding MHC-II, insulin, IL-2/IL-2RA, CTLA-4, NRAMP1, and PTPN22 (Wicker et al. 2005). For many of the disease-associated regions, however, both the causative gene(s) and mechanism(s) of susceptibility are unknown and it is possible that common disease pathways contribute to autoimmune susceptibility.

One such susceptibility region, *Idd9*, provides nearly complete protection from diabetes development when the *Idd9* ^*NOD*^ DNA segment is replaced with that derived from the B10 strain (*Idd9* ^*B10*^) (Lyons et al. 2000). Congenic mice with progressive truncations of the *Idd9* region were used to determine that at least three independent disease genes, *Idd9.1*, *Idd9.2*, and *Idd9.3*, are located within *Idd9*. The immune-signaling molecule CD137/4-1BB was identified as the candidate gene for *Idd9.3* based on amino acid variation (Lyons et al. 2000) and functional differences (Cannons et al. 2005). We developed congenic strains isolating the *Idd9.1* (Chamberlain et al. 2006; Yamanouchi et al. 2009) and *Idd9.2* (Hamilton-Williams et al. 2009) intervals to define the biological effects of these genes, and in the current study we use these and additional, novel congenic strains to fine-map the *Idd9.1* and *Idd9.2* regions and define candidate genes responsible for diabetes protection.

Diabetes protective regions on chromosome 4 that overlap *Idd9* have been characterized using congenic regions derived from the C57BL/6 (B6) and NOR strains (Stolp et al. 2012; Tan et al. 2010). *Idd11*, which partially overlaps *Idd9.1*, has been defined using NOD.B6 congenic strains, and differential expression of a novel gene (*AK005651*, also known as *1700003M07Rik*) within the region has been reported (Tan et al. 2010). A recent study has fine-mapped two other protective regions overlapping *Idd9.1* and *Idd9.2*, but not *Idd11*, derived from the NOR strain, which has a mixed genetic background derived from the NOD, B6, and DBA/2 genomes (Stolp et al. 2012). The *Idd9* region also overlaps with a B10.NOD congenic region defining *Nss1*, a gene region that influences the development of sialadenitis (Hjelmervik et al. 2007). Although the *Idd9* region does not overlap with any known human T1D susceptibility loci, it contains several genes of immunological significance and genes with variations linked to other human diseases (e.g., genes encoding Lck, MTOR, MASP2, and CD137) (Hildebrandt et al. 2009; Pu et al. 2011; Sorensen et al. 2005).

A number of studies have identified immune-related phenotypic defects in NOD mice that are corrected by the presence of either B10-, B6-, or NOR-derived alleles at the *Idd9* overlapping regions. We have shown that NOD congenic mice that carry *Idd9* ^*B10*^ alleles have restored CD8^+^ T-cell tolerance to the islet antigen islet-specific glucose-6-phosphatase catalytic subunit-related protein (IGRP) (Hamilton-Williams et al. 2010). This restored tolerance was mediated primarily by the *Idd9.2* subregion. Although CD8^+^ T-cell tolerance was restored by protective *Idd9* alleles, intrinsic expression of these alleles was required by CD4^+^ T cells and a nonlymphocyte cell type. In another study, the ability of IGRP-specific CD8^+^ T cells to induce diabetes was also reduced by *Idd9* ^*B10*^ alleles (Yamanouchi et al. 2009). Likewise, this was not due to intrinsic expression of *Idd9* genes within the CD8^+^ T cells, but was mediated by an *Idd9.1* effect that enhanced the suppressive activity of FoxP3^+^CD4^+^CD25^+^ regulatory T cells. *Idd9.3* has been found to increase the accumulation of CD137^+^ regulatory T cells, strengthening the likelihood that the amino acid variation in CD137 determined by *Idd9.3* alleles regulates T1D susceptibility (Kachapati et al. 2012). Islet-specific CD4^+^ BDC2.5 T cells expressing *Idd9* ^*B10*^ alleles were found to be less pathogenic than their NOD counterparts (Waldner et al. 2006). The *Idd9.1* ^*B10*^ region reduced the islet-specific CD8^+^ T-cell response in a TNF-α-mediated model of T1D (Chamberlain et al. 2006). The NOR-derived T1D resistance loci that overlap *Idd9.1* and *Idd9.2* have been shown to reduce the pathogenic capacity of both CD4^+^ T cells and B cells (Chen et al. 2008; Silveira et al. 2006; Stolp et al. 2012). Lastly, *Idd9* ^*B10*^ alleles were found to contribute to reducing the susceptibility of β cells within islets to CTL killing, which was linked to expression of an *Idd9* candidate gene, *Tnfr2* (Hill et al. 2007).

Given the large number of biological effects attributed to genes within the *Idd9/11* regions, further definition of the polymorphic gene content within the subregions altering disease susceptibility is warranted. In the current study, we have developed new congenic strains and refined the genetic definition of previously characterized strains to reduce the physical size of the *Idd9.1* ^*B10*^ and *Idd9.2* ^*B10*^ regions and to assess their polymorphic gene content. Using CD8^+^ T-cell tolerance and CD4^+^ T-cell gene expression as readouts, we define potential mechanisms of disease protection mediated by polymorphic genes within the *Idd9.1* ^*B10*^ and *Idd9.2* ^*B10*^ regions.

## Materials and methods

### Mice

**Fig. 1:**
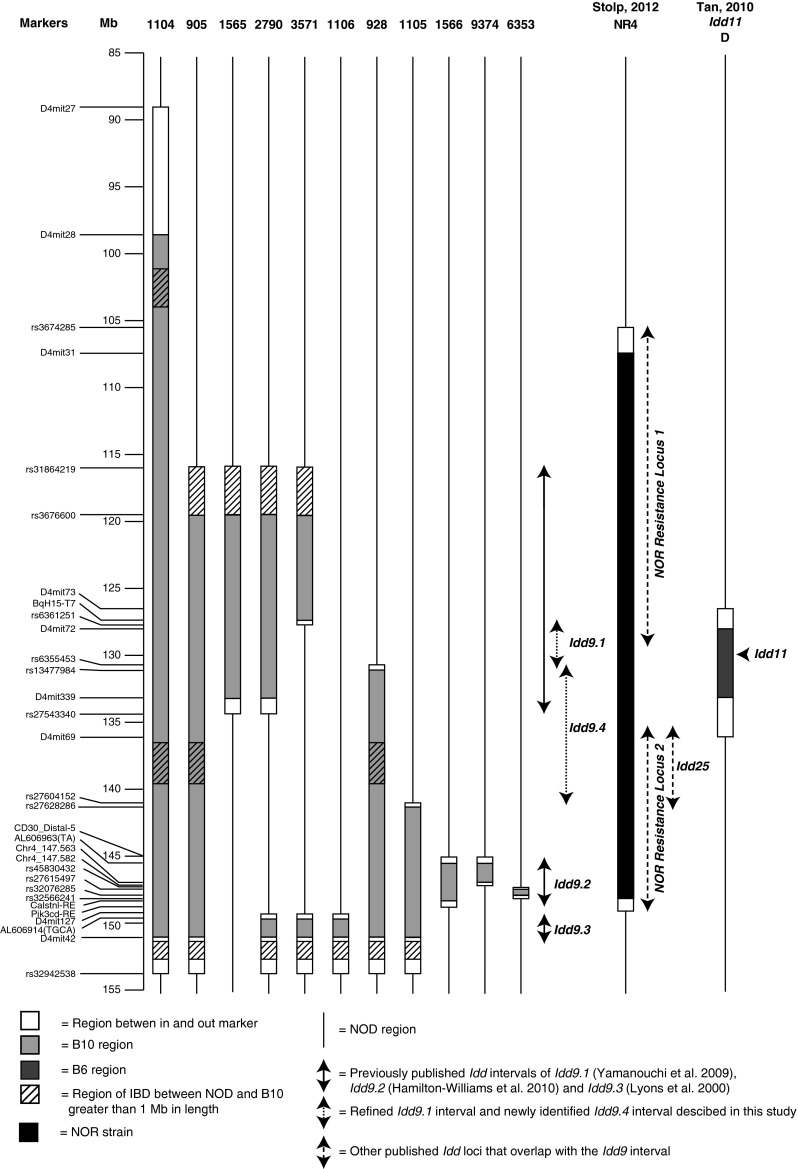
A map of the *Idd9* congenic strains used in this study. The congenic strains used to define the *Idd9.1*, *Idd9.2*, and *Idd9.4* intervals and those used in the T1D frequency studies are shown

Experimental procedures were performed according to the National Institutes of Health Guide for the Care and Use of Laboratory Animals (IACUC #09-0074). NOD/MrkTac and NOD-SCID (NOD/MrkBomTac-*Prkdc* ^*scid*^) mice were purchased from Taconic (Hudson, NY). For clarity, a diagram of the congenic strains used in this study is shown in Fig. 1. The NOD.B10 *Idd9* congenic strain line 905 (NOD.B10-*Idd9* ^*C57BL/10SnJ*^/R905MrkDvsJ) contains a continuous B10-derived DNA segment that includes T1D-protective alleles *Idd9.1*, *Idd9.2*, and *Idd9.3* (Chamberlain et al. 2006; Hamilton-Williams et al. 2010; Martinez et al. 2005). Line 905 was made by crossing line 1104 (called R28 in Lyons et al. 2000) to NOD/MrkTac and screening for recombinants that removed the proximal end of 1104. Lines 928 (not published previously) and 1565 (NOD.B10Sn-*Idd9.1* ^*C57BL/10SnJ*^/1565MrkTacJ) (Chamberlain et al. 2006; Hamilton-Williams et al. 2010; Yamanouchi et al. 2009) were derived by crossing line 905 to NOD/MrkTac to identify recombinants that removed the proximal *Idd9.1* and distal *Idd9.2* and *Idd9.3* regions, respectively. Line 2790 (*Idd9.1+Idd9.3*) was developed by combining the congenic segments present in lines 1565 and 1106 (NOD.B10Sn-*Idd9.3* ^*C57BL/10SnJ*^/1106MrkTacJ, containing *Idd9.3* and called R35 in Lyons et al. 2000). During the development of line 2790, a new recombinant was identified that removed the distal portion of the *Idd9.1* region and line 3571 was developed. Line 1105 (having a single congenic segment encompassing both *Idd9.2* and *Idd9.3*, called R11 in Lyons et al. 2000) and line 1566 (containing *Idd9.2*, NOD.B10Sn*-Idd9.2* ^*C57BL/10SnJ*^ */*1566MrkTacJ) have been previously described (Cannons et al. 2005; Hamilton-Williams et al. 2010; Lyons et al. 2000; Yamanouchi et al. 2009). The proximal end of line 1105 was defined more precisely than previously published by screening additional markers (Supplementary Table 1a). The *Idd9.2*-SCID strain was generated by crossing line 1566 and NOD-SCID mice followed by selective breeding. While making the *Idd9.2*-SCID line, we identified a recombination event that isolated the proximal portion of *Idd9.2* and line 9374 was developed. After failing to identify a recombination event that isolated the distal portion of *Idd9.2*, a different approach was taken. Line 930 (unpublished, containing the distal portion of *Idd9.2* in addition to *Idd9.3*) was backcrossed to NOD and line 6353 was developed, isolating the distal portion of *Idd9.2*. The NOD.B10 congenic line 6359 was used as the NOD control strain in the CD8^+^ T-cell tolerance and gene expression studies; line 6359 contains a small B10-derived congenic region on chromosome 1 that does not reduce diabetes incidence compared with NOD (Hunter et al. 2007).

### Genotyping

DNA extraction for genotyping and genotyping methods were described previously (Fraser et al. 2010). Primer3 (Rozen and Skaletsky 2000) was used to design primers for PCR that were then synthesized by Sigma-Genosys (Haverhill, UK). All primers designed for this study are listed in Supplementary Table 1b.

### Variant identification

The *Idd9.2* and portions of the *Idd9.1/9.4* regions have been resequenced using NOD BAC clones selected and sequenced at the Welcome Trust Sanger Institute (Steward et al. 2010) and deposited at the European Molecular Biology Laboratory (http://www.ebi.ac.uk/embl/). A list of BAC clones sequenced for each region can be found at http://www.sanger.ac.uk/cgi-bin/Projects/M_musculus/mouse_NOD_clones_TPF. BAC clones were selected based on the (then current) NCBIm37 genome build. Recent analysis of optical map data shows that the *Idd9.2* tiling path file in NCBIm37 was partly incorrect across the proximal portion represented by congenic lines 1566 and 9374 and was restructured in the new GRCm38 mouse genome build. The NOD genome along with the genomes of 17 other inbred strains of mice has also been sequenced using next-generation sequencing technology (http://www.sanger.ac.uk/resources/mouse/genomes/). Single nucleotide polymorphisms (SNPs) from the analyzed *Idd* regions were entered into T1Dbase (Hulbert et al. 2007; Smink et al. 2005) and displayed graphically using Gbrowse (Stein et al. 2002). Variant SNPs between strains were downloaded from the Mouse Phenome Database (http://phenome.jax.org/) using the datasets CGD-MDA1, Broad2, WTCHG1, and JAXSNP1. All of the annotation of the intervals can be viewed at http://www.t1dbase.org. Ensembl annotation was imported into T1Dbase. These Ensembl annotations are a merged gene set derived from the manual annotation created by the HAVANA team (Wilming et al. 2008) and the Ensembl evidence-based automatic pipeline (Curwen et al. 2004). The Ensembl/Havana merged gene set is produced using a method similar to that used for the human ENCODE project (Harrow et al. 2006).

Due to the repetitive nature of the *Idd9.2* region, direct homologous gene observation between NOD and B6 was not possible in the segmentally duplicated region. Both mouse sequences were subjected to further manual scrutiny by the HAVANA team using standard annotation rules and software described on the HAVANA website (http://www.sanger.ac.uk/research/projects/vertebrategenome/havana/). These annotations were published to the Vega website (http://vega.sanger.ac.uk/Mus_musculus/Info/Index). Nucleotide sequences of homologous genes in the B6 and NOD *Idd9.2* regions were aligned with MAFFT v6.857, variants were derived from the alignments using an “ad-hoc” Perl script, and variant consequences were obtained with SnpEff v2.0.5d based on annotations extracted from the HAVANA internal database, taking the B6 as the reference genome.

### Virus

Recombinant vaccinia virus expressing the H-2K^d^ restricted epitope VYLKTNVFL, amino acid residues 206–214 of murine IGRP (Vac-K^d^IGRP), was previously described (Hamilton-Williams et al. 2010). Mice were infected intraperitoneally (i.p.) with 1 × 10^7^ PFU of virus and CD8^+^ T-cell responses were measured in the spleen 7 days later by tetramer staining.

### Flow cytometry

CD8^+^ T cells were stained with H-2K^d^-IGRP_206-214_–PE tetramers (NIAID MHC Tetramer core facility) for 15 min at room temperature (RT) followed by staining with anti-CD8-FITC at 4 °C for 15 min. All monoclonal antibodies were obtained from either eBioscience (San Diego, CA), BioLegend (San Diego, CA), or BD Pharmingen (San Diego, CA). Cells were acquired with either a FACS Calibur or LSRII (Becton Dickinson, Mountain View, CA) and analyzed with FlowJo software (Tree Star, Inc., Ashland, OR).

### Diabetes frequency studies

**Fig. 2:**
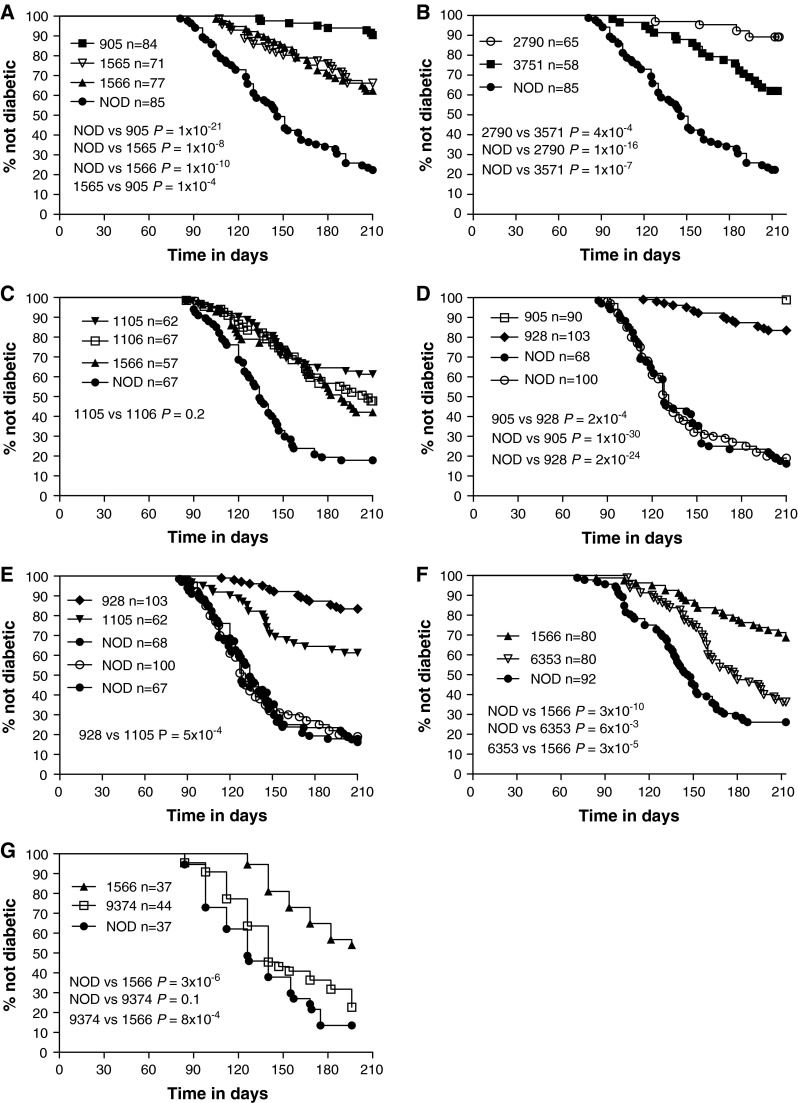
Diabetes incidence of novel *Idd9.1* and *Idd9.2* congenic strains. **a** Line 905, 1565, 1566, and NOD mice were monitored for diabetes. **b** Line 2790, 3571, and NOD mice were monitored for diabetes. **c** Line 1105, 1106, 1566, and NOD mice were monitored for diabetes. Data from lines 1106, 1566, and NOD (but not 1105) in this panel were previously published in Yamanouchi et al. (2009). **d** Line 905, 928, and NOD (×2) were monitored for diabetes. **e** Combined data from C and D. **f** Line 1566, 6353, and NOD mice were monitored for diabetes. **g** Line 1566, 9374, and NOD mice were monitored for diabetes

Female mice were tested for the presence of T1D every 7–14 days beginning at ~80 days of age by the detection of urinary glucose >500 mg/dL using Diastix (Miles, Elkhart, IN). Diabetes frequency studies were performed at Merck Research Laboratories, except for the experiment depicted in Fig. 2g which was conducted at Taconic.

### RNA extraction

For gene array analysis, CD4^+^ T cells pooled from the pancreatic lymph nodes (PcLNs) of four female 8–10-week-old mice were stained with anti-CD4, CD44, CD62L, and CD25 (BD Pharmingen) and sorted into naïve (CD44^low^ CD62L^high^ CD25^-^), activated (CD44^high^ CD62L^low^), central memory (CD44^high^ CD62L^high^), and regulatory (CD44^low^ CD62L^high^ CD25^+^) phenotype CD4^+^ T cells with a FACS ARIA (Becton Dickinson). For QRT-PCR analysis, CD4^+^ T cells were similarly sorted from spleen cells pooled from two mice per sample. Total nonlymphocyte spleen cells were prepared from SCID mice by lysis of red blood cells in ACK lysis buffer (Lonza, Walkersville, MD). Islets were isolated from the pancreas by digestion in collagenase P and hand picking as described (Gazda et al. 1997). RNA was extracted in TRIZOL reagent (Invitrogen, Carlsbad, CA). RNA (500 ng) was used to make cDNA with a High Capacity cDNA Reverse Transcription Kit with Rnase Inhibitor (Applied Biosystems, Carlsbad, CA).

### DNA array sample preparation and hybridization

The Scripps Research Institute DNA Array Core Facility performed DNA array sample preparation, hybridization, and statistical analysis on triplicate samples. Sample quality was checked with the Agilent 2100 Bioanalyzer using the RNA 6000 Pico LabChip (Agilent Technologies, Santa Clara, CA). Total RNA (5 ng) was amplified with the WT-Ovation Pico RNA Amplification System version 1.0 (NuGEN Technologies, San Carlos, CA). Post-amplification product was processed with the WT-Ovation Exon Module version 1.0 (NuGEN Technologies). Post-exon product was fragmented and labeled using the FL-Ovation cDNA Biotin Module V2 (NuGEN Technologies). Pre- and post-fragmentation products were checked on an RNA 6000 Nano LabChip (Agilent Technologies) using the mRNA Assay program. The post-fragmentation and labeling product was used for the hybridization cocktail and hybridized overnight to the Affymetrix GeneChip Mouse Exon 1.0 ST Array (Affymetrix, Santa Clara, CA) following the NuGEN FL-Ovation cDNA Biotin Module V2 protocol. Hybridization and scanning of samples to arrays was performed using standard Affymetrix protocols and reagents from the GeneChip Hybridization, Wash, and Stain Kit (Affymetrix). Chips were scanned using the Affymetrix GeneChip Scanner 3000 7G with default settings and a target intensity of 250 for scaling.

### DNA array statistical analysis

The DNA array analysis was run using XRAY software version 3.9932 (Biotique Systems Inc., Reno, NV). In order to identify genes with differential gene expression or alternative splicing between the groups, mixed-model analysis of variance was used. The input files were normalized with full quantile normalization (Irizarry et al. 2003). Background correction was established from a pool of probes stratified by CG content. Probe Summarization for each probe set was derived via application of median polish (exon RMA) to the probe scores across all input hybridizations and probe sets. A multiple-tests correction was performed using the Benjamini and Hochberg False Discovery Rate method (Benjamini and Hochberg 1995).

### QRT-PCR analysis

QRT-PCR analysis was performed using 12.5 ng of template cDNA, the primers listed in Supplementary Table 1c, with SYBR^®^ Green PCR Master Mix (Applied Biosystems) on a ABI 7900 HT fast real-time PCR system (Applied Biosystems). Expression levels were normalized to β2 m (CD4^+^ T-cell samples) or actin (SCID spleen and islet samples). Actin normalization was used for the experiment, including SCID spleen samples, as these contain a high proportion of antigen-presenting cells, which may upregulate β2 m upon activation.

### Statistical analysis

Kaplan–Meier diabetes survival curves were plotted and compared using the log-rank test. Differences in the frequency of tetramer binding cells were compared between groups via the Mann–Whitney test. Differences in gene expression were compared between groups via a *t* test. All tests were carried out using GraphPad Prism software (GraphPad Software, Inc., La Jolla, CA).

## Results

### Refinement of the *Idd9.1* genetic interval by physical mapping

To define candidate genes responsible and interpret the biological effects of the *Idd9.1* and *Idd9.2* subregions, we developed congenic lines that isolated each protective region independently. *Idd9* was originally defined by a 44.7-cM (66.4 Mb) B10-derived interval in line 1104 (Lyons et al. 2000). The proximal portion of this interval was truncated by 27 Mb, resulting in line 905 (Fig. 1). Similar to the previously published diabetes frequency of line 1104 (Lyons et al. 2000), line 905 was almost completely protected from diabetes (905 vs. NOD *P* = 1 × 10^−21^) (Fig. 2a). This indicates that line 905 still contains the entire *Idd9* region. Backcrossing line 905 to NOD, we screened for a recombinant that isolated *Idd9.1*. Line 1565 was significantly protected from diabetes when compared to NOD (*P* = 1 × 10^−8^), but was not as protected as line 905 (1565 vs. 905 *P* = 1 × 10^−4^) (Fig. 2a). An independent frequency experiment performed with line 1565 showed an almost identical level of protection from diabetes (Yamanouchi et al. 2009). This indicates that B10 alleles at the *Idd9.1* region alone (line 1565) are partially protective, but the three B10-derived *Idd9* subregions combined provide greater protection.

During the development of an *Idd9.1* and *Idd9.3* double congenic strain (line 2790), we identified a recombinant that removed the distal portion of *Idd9.1* (line 3571). Both lines 2790 and 3571 were protected from diabetes when compared to NOD (2790 vs. NOD *P* = 1 × 10^−16^, 3571 vs. NOD *P* = 1 × 10^−7^), but line 3571 was not as protected as line 2790 (*P* = 4 × 10^−4^, Fig. 2b), indicating that line 3571 had lost the protective effect of *Idd9.1*. As line 3571 and line 1106 (*Idd9.3*) were not compared in the same experiment [for reference, a previously published line 1106 incidence study is shown in Fig. 2c (Yamanouchi et al. 2009), and an independent line 1106 incidence was also previously published in Lyons et al. (2000)], it is not possible to exclude the possibility that the proximal portion of *Idd9.1* found in line 3571 contains an additional weakly protective gene. However, it is unlikely as the incidence of line 3571 and overall incidences of line 1106 were extremely similar. The difference between strains 2790 and 3571 maps the *Idd9.1* protective region to 6.249 Mb.

### Identification of *Idd9.4*

During the line 905 backcross, a recombinant was identified that removed the proximal end of the congenic region creating line 928 (Fig. 1). Line 928 was compared to line 905 (*Idd9.1*, *Idd9.2*, and *Idd9.3*) in order to determine whether the proximal portion that was removed contained a protective gene (Fig. 2d). Line 928 was protected from diabetes when compared to NOD (*P* = 2 × 10^−24^) but was not as protected as line 905 (*P* = 2 × 10^−4^). This shows that the proximal portion removed from line 928 contains a protective gene that we continue to call *Idd9.1*. Combining this incidence data with that above, our data support that the *Idd9.1* protective allele is mapped by the difference in lines 3571 and 928, which is a 2.935-Mb region.

As line 928 contained the *Idd9.2* and *Idd9.3* regions, we also compared the diabetes frequency to line 1105 (containing *Idd9.2* and *Idd9.3*). The diabetes incidence study depicted in Fig. 2d (including line 928) was conducted at Merck in 2000–2001. A second incidence study (including line 1105) shown in Fig. 2c, was also conducted at Merck in 2002–2003. As these two studies were conducted in the same animal house relatively close in time and the frequencies of diabetes in the NOD females were identical in both studies, we compared the incidences of lines 928 and 1105 (Fig. 2e). In this analysis, line 928 was more protected than line 1105 (*P* = 5 × 10^−4^). Taken together, this shows that line 928 contains an additional protective gene (*Idd9.4*) compared to line 1105, defined by the difference in lines 1105 and 928, a 10.626-Mb region (Fig. 1). Since we have not developed isolated *Idd9.1* and *Idd9.4* strains containing the 2.935- and 10.626-Mb intervals, respectively, as defined by the results of the interval truncation experiments described above, it is possible that the protection from T1D observed in line 1565 is due to the combined effect of protective alleles at both the *Idd9.1* and *Idd9.4* regions.

### Refinement of the *Idd9.2* genetic interval by physical mapping

*The Idd9.2* region was originally defined as the difference between lines 1105 (formally R11) and 1106 (formally R35) (Lyons et al. 2000), a 5.6-Mb region that includes the candidate genes *Cd30* (since renamed *Tnfrsf8*), *Tnfr2* (since renamed *Tnfrsf1b*), and *Mtor*. In the subsequent diabetes frequency study depicted in Fig. 2c, line 1105 was not significantly more protected than line 1106 (*P* = 0.20), although line 1105 trended to a slightly lower diabetes frequency at 210 days. Despite this and based on the earlier data, the *Idd9.2* interval was isolated and reduced to a 3.9-Mb region defined by strain 1566, containing more than 40 genes (Hamilton-Williams et al. 2010; Yamanouchi et al. 2009). Importantly, line 1566 does not contain *Cd30* and *Tnfr2*. Consistent with our previously published finding (Yamanouchi et al. 2009) and shown here in three independent, previously unpublished, diabetes frequency studies (Fig. 2a, f, g), line 1566 was significantly protected from diabetes compared with NOD mice (*P* = 1 × 10^−10^, *P* = 3 × 10^−10^, and *P* = 3 × 10^−6^), demonstrating that this region retains *Idd9.2*.

**Fig. 3:**
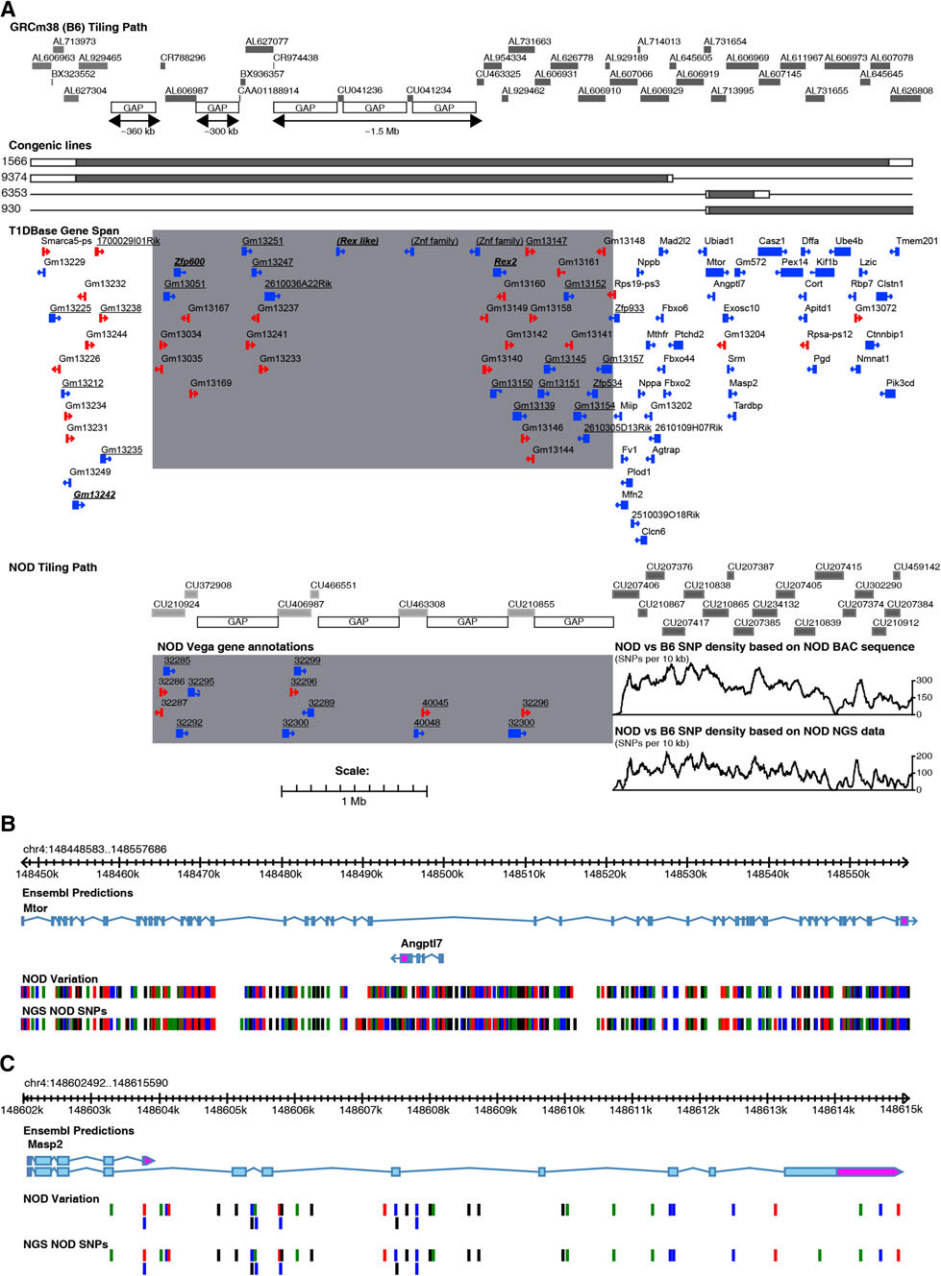
Map of the congenic strains, gene content, and SNP density of the *Idd9.2* region based on GRCm38 genome build. **a** The NOD.B10 congenic regions within strains 1566, 9374, 6353, and 930 are shown; the *grey bars* indicate B10-derived DNA and the *white bars* indicate the regions between the “in” and “out” markers. The gene content is displayed in the T1Dbase Gene Span tract (based on GRCm38 genome build), with known genes shown in blue and pseudogenes in red. Zinc finger family members are *underlined* and those with high homology to *Rex2* are also *bold* and *italicized*. The *grey box* contains the region with low sequence homology between the NOD and B6 sequence. The NOD tile path track represents the sequenced NOD BAC clones, the *light grey* clones are in the region with low sequence homology to B6, and their exact position is difficult to determine. The NOD Vega gene annotations are shown for these clones with their Vega database gene identifier, which has the prefix OTTMUSG000000. The SNP density tracks are shown for the region containing high sequence homology for BAC sequence and NGS data; display is SNPs per 10 kb. **b** Detailed SNP track covering the *Mtor* gene region. **c** Detailed SNP track covering the *Masp2* gene region. The B6/NOD SNPs tracks represent the location of the polymorphic NOD/B6 SNPs; *black*, *red*, *blue*, and *green lines* represent G, T, C, and A NOD alleles, respectively. Note that where multiple SNPs are located close together the lines in the B6/NOD SNPs track may represent more than one SNP

To reduce the number of potential gene candidates, two new congenic strains (lines 6353 and 9374) were produced that contained smaller regions of *Idd9.2* as defined by line 1566 (Figs. 1, 3a). Line 9374 contains the proximal half of the *Idd9.2* region, including *Fbxo2* (encoding F-box protein 2), which has been implicated to have a role in diabetes progression (Kodama et al. 2008). Line 6353 contains 10 genes, including *Mtor* (encoding mammalian target of rapamycin [mTOR], diagrammed in Fig. 3b) and *Masp2* (encoding mannan-binding lectin serine peptidase 2 [MASP-2], diagrammed in Fig. 3c), which both have functions in the immune system. Unfortunately, despite screening for recombination events within *Idd9.2* in ~500 mice, a recombinant mouse having the entire distal portion of the *Idd9.2* region in isolation was not identified. The T1D frequencies of lines 9374 and 6353 were compared to those of NOD and 1566 mice (Fig. 2f, g). Line 6353 was significantly more protected than NOD (*P* = 6 × 10^−3^) but less protected than line 1566 (*P* = 3 × 10^−5^). Line 9374, however, had a disease incidence that was indistinguishable from that of control NOD mice (*P* = 0.1). This supports the hypothesis that more than one protective gene accounts for the protection provided by line 1566, including a gene encoded within line 6353. It remains a possibility that line 9374 contains a T1D protective gene that is only protective when a second distal *Idd9.2* gene is present. Alternatively, an additional gene may be present in the region between lines 9374 and 6353 or distal to line 6353.

### Mapping the *Idd9.2* genetic interval by assessment of CD8^+^ T-cell tolerance to islet antigen IGRP

**Fig. 4:**
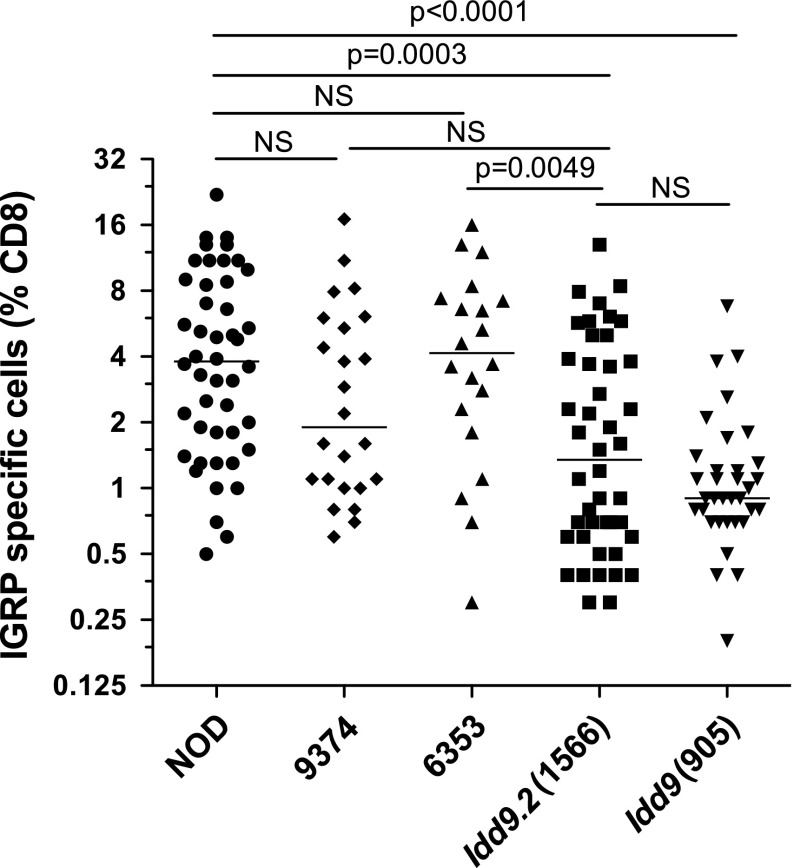
IGRP-specific CD8^+^ T-cell responses in *Idd9.2* congenic substrains. NOD, 9374, 6353, 1566 (*Idd9.2*), and 905 (*Idd9*) mice were infected with Vac-IGRP and the frequency of IGRP-specific CD8^+^ T cells in the spleen was determined. Pooled data from six similar experiments are shown. *Horizontal line* depicts median value

Previously, we showed that protection from diabetes in *Idd9* congenic mice correlates strongly with restored CD8^+^ T-cell tolerance to islet antigens (Hamilton-Williams et al. 2007, 2010; Martinez et al. 2005). Of the three individual *Idd9* subregions, *Idd9.2* provided the strongest independent effect on CD8^+^ T-cell tolerance and *Idd9.1* had no effect (Hamilton-Williams et al. 2010). We therefore tested whether either of the lines having a portion of the *Idd9.2* region present in line 1566, line 9374 or 6353, had a reduced frequency of IGRP-specific CD8^+^ T cells. NOD, *Idd9* (905), *Idd9.2* (1566), 9374, and 6353 mice were infected with Vac-K^d^IGRP to expand the population of any IGRP-specific CD8^+^ T cells that were present (Fig. 4). As expected, NOD mice had a high frequency of IGRP-specific CD8^+^ T cells, which was significantly reduced in the *Idd9* (*P* < 0.0001) and *Idd9.2* strains (*P* = 0.0003). However, 6353 mice were not significantly different from NOD, and 9374 mice were intermediate between NOD and 1566 (not significantly different from either strain). We concluded that line 9374 contains a gene that has a small effect on CD8^+^ T-cell tolerance induction; the effect on tolerance could reflect the activity of a gene that contributes to T1D protection only when a second gene located in the distal region of line 1566 is also present. However, as neither line 6353 nor line 9374 gave as strong a phenotype as the full *Idd9.2* region contained in 1566 mice, we continued to use the *Idd9.2* region defined by the 1566 strain in our subsequent expression and sequence analyses.

### Haplotype and sequence analysis of *Idd9.1* and *Idd9.4*

**Fig. 5:**
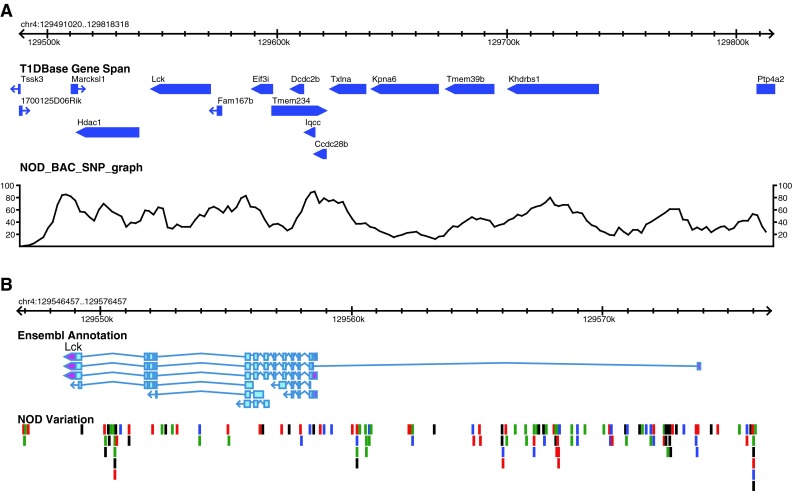
Sequence analysis of the *Idd9.1* region between 129.491 and 129.818 Mb, where B10 and B6 are IBD, and compared to NOD. **a** The gene content is displayed in the T1Dbase Gene Span tract (based on GRCm38 genome build) and the SNP density is displayed as SNPs per 10 kb. **b** Detailed SNP track covering the *Lck* gene region. The B6/NOD SNPs tracks represent the location of the polymorphic NOD/B6 SNPs; *black*, *red*, *blue*, and *green lines* represent G, T, C, and A NOD alleles, respectively. Note that where multiple SNPs are located close together, the *lines* in the B6/NOD SNPs track may represent more than one SNP

We next performed haplotype and sequence analyses of the *Idd9.1/9.4* and *Idd9.2* regions with the aim of identifying potential SNPs that could be the causative polymorphisms differing between the NOD and B10 strains and to help reduce the number of potential candidate genes within the regions. SNP data for all available strains was downloaded (http://phenome.jax.org/pub-cgi/phenome/mpdcgi) and compared to identify regions of sequence variation and regions that are identical by descent (IBD) between the B10 and NOD strains. Of the 123 strains with SNP data available, the B10 haplotype at *Idd9.1/9.4* was fully shared only by the strains BDP/J, P/J, SEA/GnJ, and TKDU/DnJ (Supplementary Table 1). Notably, unlike much of the genome, this is a region that is not IBD throughout for the B10 and B6 strains. Based on this SNP analysis, the B10 strain does not share its full haplotype at the *Idd9.1/9.4* region with any of the 18 strains for which full sequence data are available (Supplementary Table 2). However, there are smaller regions of IBD shared between B10 and the 18 strains with complete genomic sequence (Supplementary Table 2) and this sequence can be used for haplotype and sequence analyses. For example, within the *Idd9.1* region, B6 and B10 are identical across 313 kb from 129.495 to 129.808 Mb (77 identical SNPs, Supplementary Table 2); this region contains 17 genes, including the candidate gene *Lck*. A sequence comparison with the NOD strain is shown in Fig. 5 and Supplementary Table 3. Although no polymorphisms in the predicted amino acid sequence encoded by *Lck* were found, numerous polymorphisms located in noncoding regions of the gene were present that could potentially alter *Lck* expression. Coding changes in several other genes (Supplementary Table 3a) as well as variation in a noncoding gene (Supplementary Table 3b) in this highly polymorphic region were also present, although none of the genes is a compelling candidate gene based on its known immune function. *Idd9.1* overlaps the distal portion of the *NOR Resistance Locus 1* (Fig. 1), and the B10 and NOR haplotypes are IBD in this overlapping region (Supplementary Table 2) and could therefore share the same T1D protective allele in this region.

For the 10.626-Mb *Idd9.4* susceptibility region, NOD and B10 have the identical haplotype for the 2.592-Mb region, from 137.091 to 139.683 Mb (Fig. 1; Supplementary Table 2), excluding this region from containing the causative gene for *Idd9.4*. Only small stretches of the remaining 8 Mb of *Idd9.4* are potentially IBD between NOD and B10. There are portions of *Idd9.4* where strains with a complete genomic sequence and the B10 strain are IBD and differ from NOD such as where B10 has 1,270 identical SNPs compared to NZO/HlLtJ at 134.742–139.683 Mb (Supplementary Table 2). These overlapping regions could be interrogated for sequence variants as described for the *Lck* region above. However, defining potential candidate genes in this region would best be approached by first reducing the size of *Idd9.4* with additional congenic strains separating portions of the ~8-Mb congenic segment that are highly polymorphic between the NOD and B10 strains. It is of note that throughout much of the B10-defined *Idd9.4* region that overlaps the NOR-defined *Idd25* region, which comprises the proximal portion of the *NOR Resistance Locus 2* (Fig. 1; Supplementary Table 2), the two strains are generally not IBD except where they are both also IBD with the NOD strain. An exception is 136.439–137.073 Mb, where NOR and B10 are IBD and both differ from NOD, highlighting a region that could contain a T1D susceptibility gene shared by *Idd9.4* and *Idd25*. Since the B6 strain is IBD with NOR and B10 in the 136.439–137.073-Mb region, a complete sequence comparison can be made (data not shown).

### Haplotype and sequence analysis of *Idd9.2*

As all 775 B6 and B10 SNPs were found to be IBD throughout the *Idd9.2* region (data not shown), we compared NOD and B6 sequences across this region (Fig. 3a). The proximal portion of the *Idd9.2* region contained a high degree of repetitive sequences and contains several sequence breaks in the B6 assembly. As a result we were unable to generate a continuous NOD sequence through the proximal portion of *Idd9.2* and were unable to compare the NOD and B6 sequences for the proximal region indicated on Fig. 3a. Homologous genes between NOD and B6 that could not be confidently identified were annotated cautiously. In particular, the sequence distal of *Gm13051* becomes increasingly divergent between B6 and NOD and breaks down almost completely distal of *Zfp600*. A very high SNP density (up to 400 SNPs per 10 kb) was found throughout the entire distal *Idd9.2* region, indicative of an ancient haplotypic divergence (Yang et al. 2011); thus, all genes in the region are potentially functionally polymorphic between B6 and NOD. Therefore, it is not possible to narrow the number of candidate causative genes in the *Idd9.2* region by sequence analysis alone. A list of NOD/B6 polymorphisms predicted to change the coding sequence of known genes is found in Supplementary Table 3c. Polymorphisms were also found in a number of noncoding antisense RNA and long intergenic noncoding RNA sequences (listed in Supplementary Table 3d).

The second aim of the sequence analysis was to determine the exact boundaries and gene content of the new strains 9374 and 6353. The markers that define the proximal and distal boundaries of line 9374 are listed in Supplementary Table 1a. Line 6353 has a maximum congenic interval of 0.432 Mb and the proximal “in” marker of the line 6353 recombination event is within the 7–8-intron of the 58-exon *Mtor* gene, and the “out” marker is upstream of *Mtor*. Therefore, the highly polymorphic *Mtor* (Fig. 3b) remains a candidate for the protective effects of line 6353 along with the genes *Angptl7*, *Exosc10*, *Srm*, *Masp2*, *Tardbp*, *Gm572*, *Gm3487*, *Gm3492*, and *Casz1* and the pseudogene *Gm13204*. Both the mTOR and MASP-2 proteins have functions associated with the immune system and are therefore the strongest candidates within line 6353 (Sorensen et al. 2005; Thomson et al. 2009). However, we note that the entire congenic region in line 6353 has extensive noncoding variation (Fig. 3a) that could cause allele-specific mRNA and protein expression differences, and there are amino acid-changing SNPs present in *Exosc10*, *Masp2*, and *Casz1* and variation resulting in a frame-shift in *Gm572* (Supplementary Table 3c). As NOR and B10 are IBD throughout the *Idd9.2* region (data not shown), it is probable that the T1D protection conferred by the distal portion of the *NOR Resistance Locus 2* is attributable to the same non-NOD alleles (Stolp et al. 2012).

### Identification of differentially expressed genes in the *Idd9* congenic regions

We have observed that intrinsic expression of *Idd9* genes within CD8^+^ T cells was not required for protection from the loss of CD8^+^ T-cell tolerance, but rather expression within CD4^+^ T cells was required (Hamilton-Williams et al. 2010). Considerable evidence from others also implicates CD4^+^ T cells as playing a role in protection mediated by *Idd9* (Chen et al. 2008; Waldner et al. 2006; Yamanouchi et al. 2009). Therefore, we performed a global gene expression analysis of *Idd9* versus NOD CD4^+^ T cells to identify potential candidate genes within *Idd9.1/9.4*, *Idd9.2*, and *Idd9.*3. CD4^+^ T cells with an activated phenotype (CD44^high^, CD62L^low^) were sorted from the PcLNs of NOD (line 6359) and *Idd9* (line 905) mice. Activated cells from the PcLN were used to obtain cells enriched for those activated by islet antigens. Exon arrays were used to allow detection of alternatively spliced gene isoforms (data deposited in GEO, accession No. GSE35482). This analysis resulted in the identification of 80 differentially expressed known genes (fold change >1.4, adjusted *P* value <0.05). However, most changes (77/80) were small and only three genes (*Akr1e1*, *Rex2*, and *Jun*) had a fold change >2.0. Analysis was also performed on the Affymetrix extended probe set (including provisional genes and long coding mRNAs) resulting in an additional 51 differentially expressed probes. A list of all differentially expressed genes and probes is found in Supplementary Table 4.

**Fig. 6:**
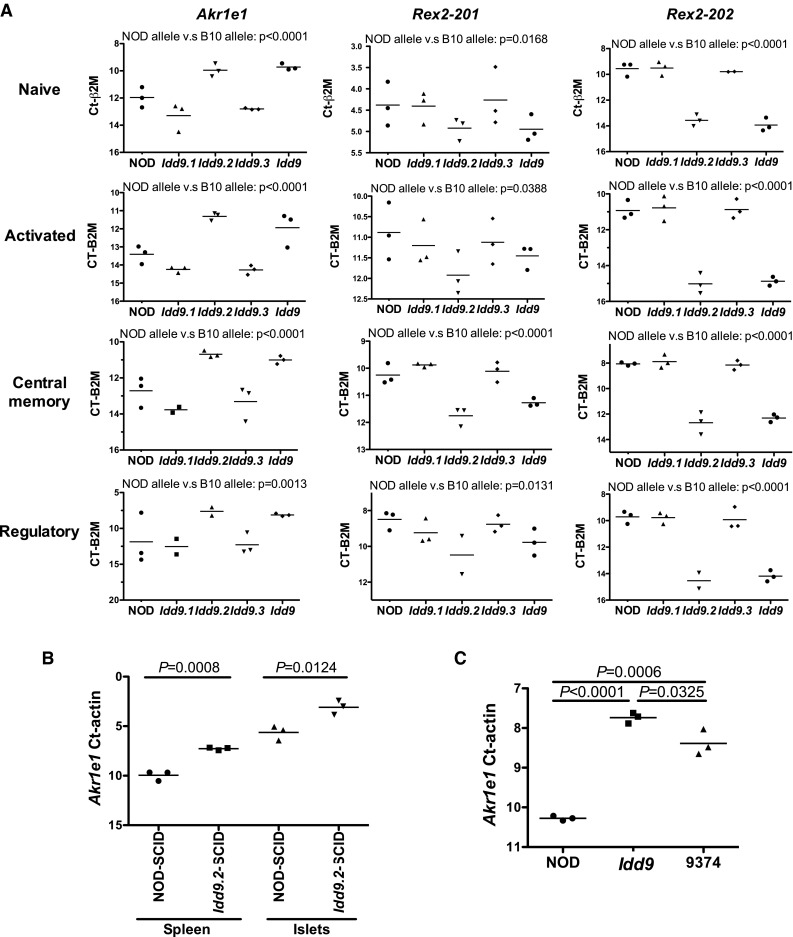
Gene expression differences dependent on *Idd9.2*. **a** Naïve, activated, central memory, and regulatory CD4^+^ T cells were sorted from splenocytes of NOD, *Idd9.1* (strain 1565), *Idd9.2* (strain 1566), *Idd9.3* (strain 1106), and *Idd9* (strain 905) mice (each point represents cells isolated from splenocytes pooled from two mice). QRT-PCR was performed and gene expression relative to β2 m is plotted. For increased statistical power, combined strains with the NOD allele at *Idd9.2* (NOD, *Idd9.1* and *Idd9.3*) were compared with combined strains with the B10 alleles at *Idd9.2* (*Idd9.2* and *Idd9*). **b** *Akr1e1* expression (exons 8 and 9) relative to actin was measured in total spleen cells and purified islets from NOD-SCID and *Idd9.2*-SCID mice. **c** Total CD4^+^ T cells were isolated from the spleens of NOD, *Idd9*, and 9374 mice and *Akr1e1* expression (exons 8 and 9) relative to actin was measured. *Line* represents mean value

In this analysis, the two most differentially expressed known genes were *Akr1e1* (encoding aldo-keto reductase family 1, member E1), which was highly upregulated, and *Rex2* (encoding reduced expression 2), which was highly downregulated in *Idd9* CD4^+^ T cells. *Rex2* is a zinc finger family member contained within the line 9374 congenic interval. The B6 proximal portion of *Idd9.2* contains no fewer than 21 zinc finger family loci (including *Rex2*, underlined genes in Fig. 3a), several of which appear to be pseudogenes. *Akr1e1* is located on chromosome 13. In order to test whether the *Idd9.2* haplotype modulated expression of these two genes, CD4^+^ T-cell subsets were isolated from the spleens of NOD, *Idd9.1*, *Idd9.2*, *Idd9.3*, and *Idd9* mice. Splenic cells were used rather than PcLN in the QRT-PCR analysis as this provided enough material to analyze gene expression without prior amplification of the RNA. *Akr1e1* and *Rex2* expression levels were analyzed by QRT-PCR (Fig. 6a). *Akr1e1* expression in naïve (CD44^low^ CD62L^high^ CD25^−^), activated (CD44^high^ CD62L^low^), central memory (CD44^high^ CD62L^high^), and regulatory (CD44^low^ CD62L^high^ CD25^+^) phenotype CD4^+^ T cells was significantly higher in *Idd9.2* and *Idd9* mice than in NOD, *Idd9.1*, or *Idd9.3* mice. Two splice forms of *Rex2* are currently annotated: *Rex2-201* and *Rex2-202*. Expression of *Rex2-202* was significantly lower in all CD4^+^ T-cell subsets tested in *Idd9.2* and *Idd9* mice compared with NOD, *Idd9.1*, and *Idd9.3* mice (*P* < 0.0001, Fig. 6a). A second primer set predicted to amplify *Rex2-201* was most significantly changed in memory phenotype CD4^+^ T cells (*P* < 0.0001, Fig. 6a) and changed to a lesser degree in the other subsets. This confirmed that expression of both *Akr1e1* and *Rex2* was strictly dependent on *Idd9.2* genotype. Differential expression of *Akr1e1* has also been observed, comparing B-cell mRNA obtained from NOD mice and NOD mice congenic for the NOR-derived region on chromosome 4 that includes *NOR Resistance Locus 2* that is IBD with B10-derived *Idd9.2* (Stolp et al. 2012).

As *Idd9* expression was determined to be required on nonlymphocyte cells as well as CD4^+^ T cells (Hamilton-Williams et al. 2009), we also compared *Akr1e1* expression in RNA isolated from *Idd9.2*-SCID- and NOD-SCID-derived spleen cells and from islets. *Akr1e1* expression was significantly higher in *Idd9.2*-SCID splenocytes than in NOD-SCID splenocytes (*P* = 0.0002, Fig. 6b). Islets were isolated from the same NOD-SCID and *Idd9.2*-SCID mice, and *Akr1e1* expression was found to be significantly higher in *Idd9.2*-SCID islets than in NOD-SCID islets (*P* = 0.0368). We also tested whether *Akr1e1* expression was regulated by a gene within the 9374 congenic strain. *Akr1e1* expression was significantly higher in CD4^+^ T cells purified from line 9374 spleens than in cells from NOD spleens (*P* = 0.0006, Fig. 6c), indicating that this region is responsible for the *Akr1e1* expression difference.

**Fig. 7:**
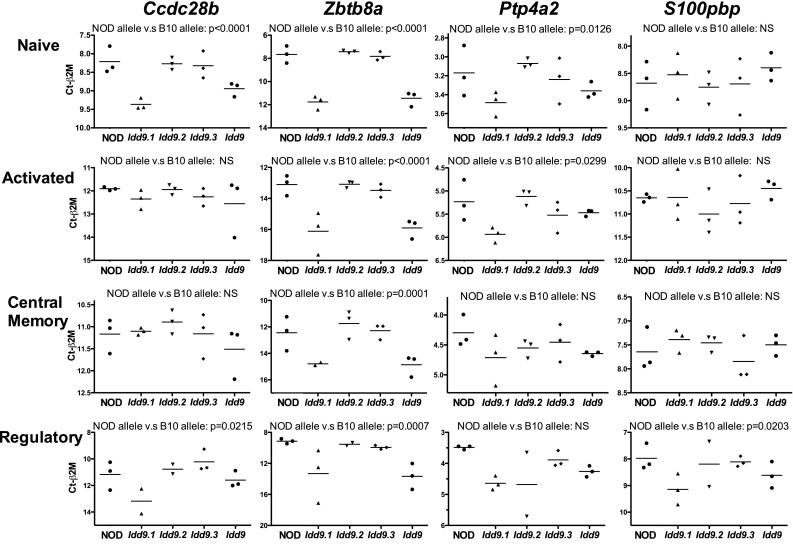
Gene expression differences in *Idd9.1* candidate genes in CD4^+^ T cells. Naïve, activated, central memory, and regulatory CD4^+^ T cells were sorted from splenocytes of NOD, *Idd9.1* (strain 1565), *Idd9.2* (strain 1566), *Idd9.3* (strain 1106), and *Idd9* (strain 905) mice (each *point* represents cells isolated from splenocytes pooled from two mice). QRT-PCR was performed and gene expression relative to β2 m is plotted. For increased statistical power, combined strains with the NOD allele at *Idd9.1* (NOD, *Idd9.2*, and *Idd9.3*) were compared with combined strains with the B10 alleles at *Idd9.1* (*Idd9.1* and *Idd9*)

The microarray gene expression analysis also resulted in the identification of a number of genes located within the *Idd9.1* region (*Ccdc28b*, *Ptp4a2*, *S100pbp*, *Rbbp4*, and *Zbtb8a*), which were studied further. QRT-PCR analysis of these genes was performed on the same CD4^+^ T-cell spleen samples as were *Rex2* and *Akr1e1*. *Ptp4a2*, *Rbbp4*, and *S100pbp* had putative differential expression of individual exons (splice variants); therefore, primers were designed to amplify the affected regions. Significant differences in the expression of *Ccdc28b*, *Zbtb8a*, *Ptp4a2*, and *S100pbp* were found in at least one of the CD4^+^ T-cells subsets examined (Fig. 7). Expression of *Rbbp4* was not found by QRT-PCR analysis to be significantly different between the strains (data not shown). *Ccdc28b*, *Ptp4a2*, and *Zbtb8a* are all located within the 2.935-Mb *Idd9.1* region shown in Fig. 1. Only *Ccdc28b* is located within the region of *Idd9.1* that overlaps with the *NOR Resistance Locus 2* (Fig. 1; Supplementary Table 2), and this gene was also reported to be differentially expressed in B cells (Stolp et al. 2012).

## Discussion

In this study we have developed several new congenic strains with the aim of defining and reducing the size of the *Idd9.1* and *Idd9.2* intervals. Analysis of the new strains has revealed that there is more than one gene affecting diabetes frequency within both regions. We have differentiated *Idd9.1* to a 2.935-Mb region and designated *Idd9.4* to a 10.626-region. The 3.9-Mb *Idd9.2* region was shown to have at least two genes influencing disease susceptibility, with one gene localized to a 0.432-Mb interval. Sequence analysis of the *Idd9.1/4* and *Idd9.2* regions revealed a high degree of variation between the B10 and NOD strains, which is consistent with these regions providing T1D susceptibility. The only observed relatively large region of IBD between the B10 and NOD strains was a 2.592-Mb region in *Idd9.4*, thereby making nearly all of the genes in the regions studied potential causal genes based on sequence variation.

The B10-derived *Idd9.1/9.4/9.2* regions overlap (Fig. 1) with the 6.9-kb B6-derived *Idd11* region (Tan et al. 2010) and with two NOR-derived regions that protect from T1D (Stolp et al. 2012). Although the NOD, B6, B10, and NOR strains have unique haplotypes overall at the distal end of chromosome 4 (McClive et al. 1994 and Supplementary Table 2), the use of a dense SNP map allows for the definition of regions where two or more strains are IBD. For example, the B6, B10, and NOR strains are all IBD and differ from NOD at the 6.9-kb *Idd11* region that is contained within *Idd9.1*, but this region has been excluded from contributing to the T1D protection provided by *NOR Resistance Locus 1* (Stolp et al. 2012; Tan et al. 2010), and B10 and NOR are not IBD with B6 in much of the remainder of the *Idd9.1* region (Supplementary Table 2). Given this, although it is possible that the T1D-susceptibility *Idd9.1* and *Idd11* genes are identical, an alternative explanation is that one or more of the many gene variants causing amino acid changes or differential gene expression of coding or noncoding genes between the NOD and B10 strains that differ from NOD/B6 variation in this region is responsible for the T1D protection observed from the B10 *Idd9.1* haplotype. The *Idd9.4* region overlaps with a newly identified NOR-derived protective region called *Idd25* as well as *NOR Resistance Locus 2* (Stolp et al. 2012). B10 and NOR are IBD for part of this region (136.439–138.545 Mb), although this partly overlaps with the region where NOD and B10 are IBD, leaving the polymorphic 136.439–137.073-Mb region that contains the complement genes *C1qa*, *C1ab*, and *C1qc*, where a common causative gene could be located. Alternatively, the B10 strain may contain a unique protective allele in the region of *Idd9.4* that is not IBD with NOR. Finally, B10 and NOR are IBD in the overlapping region encompassing *Idd9.2* and the distal portion of *NOR Resistance Locus 1.*

*Lck* has previously been proposed as a candidate gene for the *Idd9.1* region given its central role in cell signaling. We found that *Lck* remains within the 2.935-Mb *Idd9.1* region and is a strong candidate for contributing to the protective effect of *Idd9.1*. Other candidates identified include *Ccdc28b* (*coiled coil domain containing 28B*, known as *MGC1203* in humans) and *Zbtb8a* (*zinc finger and BTB domain containing 8a*). A difference in *Zbtb8a* and *Ccdc28b* expression between the NOD and B10 alleles was previously reported in the salivary glands in a model of sialadenitis (Hjelmervik et al. 2007). A difference in *Ccd28b* expression was also reported between the NOD and NOR (IBD with B10 at *Ccd28b*) alleles in B cells (Stolp et al. 2012). Differentially expressed *Ptp4a2* (*protein tyrosine phosphatase 4a2*, also known as *protein-tyrosine phosphatase of regenerating liver 2* or *PRL-2*) is a widely expressed gene (Dumaual et al. 2006) and is found overexpressed in some cancers (Hardy et al. 2010). We note that it is likely the gene expression differences between the highly polymorphic NOD and B10 *Idd9.1/9.4* and *Idd9.2* regions are greatly underestimated in our study due to insufficient sensitivity of the microarray platform and/or to the limited number of tissues and activation conditions tested. For example, *Mtor* and *Ephb2* expression in B cells having the NOR allele at these genes (in a region where NOR and B10 are IBD) differs from the expression in B cells having the NOD allele (Stolp et al. 2012). Changes in the noncoding, potentially regulatory, regions of many of the highly polymorphic genes in this region (including *Mtor*) could alter gene expression more prominently in one cell type or another. Changes in the multiple genes associated with T1D protection in this region of chromosome 4 are likely related to the changes in T- and B-cell phenotypes linked by us and others to these regions, but attributing T1D causality to any particular cellular phenotype within a subregion can only be speculative. Some of the T1D causal gene variants that are broadly expressed could influence both T- and B-cell physiology, and the overall finding from the fine-mapping conducted in the current study as well as that by Stolp and colleagues is that multiple genes within the region function together to provide the strongest alteration of multiple cellular phenotypes and T1D protection.

Variation in the rate of recombination across different genomic regions is a limitation of localizing disease genes by the congenic strain mapping approach. For example, a recombinant mouse having the entire distal *Idd9.2* congenic region was not identified in the current study. Without results from such a congenic strain, we cannot exclude that the *Idd9.2* region represented in line 9374 contains a gene that alters only diabetes susceptibility when it is expressed in combination with another gene located in the distal portion of *Idd9.2*. Interestingly, line 9374 did improve CD8^+^ T-cell tolerance to the islet antigen IGRP, supporting this possibility. Although the line 9374 diabetes study had a smaller group size (*n* = 44) compared to that of the line 6353 study (*n* = 80), and therefore had less power to detect a small individual effect on the frequency of diabetes as compared to the NOD strain, it was clear that line 9374 lacked the T1D protection mediated by the full *Idd9.2* congenic interval (line 1566).

Another limitation of the congenic mapping approach is the possibility that that multiple genes within the congenic interval modulate diabetes incidence with opposing affect. For example, a B10-derived diabetes susceptibility allele *Idd5.4* was discovered distal to the *Idd5.2* region that increased diabetes incidence (Hunter et al. 2007). The presence of such a gene could mask the presence of another protective gene in the same interval.

Sequencing of the *Idd9.2* region was complicated by the fact that the proximal portion of *Idd9.2*, as defined by line 1566, is highly duplicated, which has resulted in difficulties in providing a contiguous reference genome sequence. The success in selecting NOD BAC ends for sequencing across the proximal portion was dependent upon there being significantly high homology between the B6 and NOD mice to confidently position NOD BAC end sequences to the reference genome (Steward et al. 2010). The relatively poor sequence coverage of the proximal portion of *Idd9.2* in B6 and NOD can be explained by the repetitive nature of this region and the likely occurrence of interstrain structural variation that this causes. The existence of such divergent regions of the mouse genome within inbred strains of laboratory mice reflects the segregation of haplotypes from at least three subspecies of *Mus musculus* that diverged hundreds of thousands of years ago (Wade et al. 2002).

The entire distal portion of *Idd9.2*, including the 0.432-Mb region represented by line 6353, contains a high density of divergent SNPs when NOD and B6 sequences are compared. Two genes with known immune system function are contained in the line 6353 B10-derived congenic region, *Mtor* and *Masp2*. mTOR is a central signaling kinase that receives input from insulin, growth factors, and mitogens as well as sensing nutrient levels and redox status and then regulates cell growth and proliferation (Zoncu et al. 2011). The mTOR inhibitor rapamycin is an immunosuppressant, inhibiting effector T-cell proliferation as well as having diverse effects on antigen-presenting cells and regulatory T cells (reviewed in Thomson et al. 2009). Several variant SNPs were found within the introns of the *Mtor* gene, which could hypothetically alter expression (Fig. 3b). *Idd9*-dependent variation in mTOR protein expression was not detected by flow cytometry in naïve or activated CD8^+^ T cells (data not shown); however, this does not preclude that expression differences may occur in CD8^+^ T cells following other types of activation or in other cell types such as B cells (Stolp et al. 2012). MASP2 is a circulating blood protein related to the classical complement pathway protein C1. MASP2 is activated by the binding of pathogen-derived carbohydrates, which then allows cleavage of complement proteins C4 and C2, producing active C3 (reviewed in Sorensen et al. 2005). A known mutation in human MASP2 was associated with susceptibility to severe infections and autoimmunity (Stengaard-Pedersen et al. 2003). We detected four nonsynonymous SNPs that varied in the *Masp2* coding region (Supplementary Table 3c; Fig. 3c). Three of the four amino acid changes were nonconservative, supporting *Masp2* as a candidate gene contributing to *Idd9.2*. There are also a large number of noncoding changes in *Masp2* that could alter its expression (Fig. 3c). Although expression differences were not detected for most genes in the *Idd9.2* region, including *Mtor* and *Masp2*, the expression variation could be underestimated as discussed above.

The proximal portion of *Idd9.2* represented by line 9374 contained the gene *Rex2*, which had significant differential expression between the NOD- and B10-derived alleles, particularly in the splice variant *Rex2-202*. This may be due to a SNP or SNPs in the *Rex2* gene altering splicing sites or affecting the binding of intronic or exonic splicing enhancers or silencers. *Rex2* was identified as a cDNA expressed in teratocarcinoma cells with reduced expression following treatment with retinoic acid (Faria et al. 1998). It contains homology to zinc-finger-containing proteins and is therefore a putative DNA binding protein (Faria et al. 1998). We also identified the gene *Akr1e1*, expression of which was regulated by a gene found within the line 9374 congenic region. Akr1e1 is an enzyme involved in glycogen metabolism. Therefore, it is possible that *Rex2* modulates the transcription of *Akr1e1*.

Sequence analysis of the *Idd9.2* distal region and the *Idd9.1/9.4* regions identified a number of SNPs and larger sequence variations in noncoding RNAs. Some noncoding RNAs are believed to regulate gene expression, possibly by association with chromatin-modifying complexes (Ng et al. 2012). Therefore, it is possible that the observed variations in noncoding RNAs could alter their function and T1D susceptibility.

Our analysis shows the value of in-depth haplotype analysis. While the B10 strain has not been fully sequenced, by locating regions of IBD between B10 and another mouse strain for which full NGS data are available, we were able to identify variant SNPs in the region. Furthermore, regions of IBD between NOD and B10 can be identified and excluded and regions of commonality between the NOR, B6, and B10 protective regions could be assessed.

These studies demonstrate the difficulty of mapping disease susceptibility loci in complex genetic diseases, especially when it is possible that both protective and susceptible alleles are in linkage disequilibrium (Hunter et al. 2007). We and others have found that highly protective loci identified in segregation analyses and then confirmed with congenic strains having large regions from the parental strain are almost always ultimately shown to result from multiple disease genes (Fraser et al. 2010; Lyons et al. 2000; Serreze et al. 1998; Wicker et al. 2004). Many of these genes with small individual effects on disease can be localized by refining congenic intervals as shown in the current study. However, even when defined to less than 0.5 Mb, as done for line 6353, a congenic region still contains multiple polymorphic genes associated together as an ancient haplotypic block (Yang et al. 2011). Although genes with known immunological functions usually become the likely candidate genes, most of the genes encode proteins not previously associated with autoimmunity. It also remains possible that even in a region as small as that in 6353, there is more than one causal gene.

Nevertheless, genetic mapping studies such as this have resulted in the identification of clear single-gene candidates (Fraser et al. 2010; Jordan et al. 2011). Haplotype mapping analysis has been used to identify a key SNP in the *Ctla4* gene, which was then further verified as a causative polymorphism responsible for *Idd5.1* (Araki et al. 2009). Similarly, haplotype mapping was of key importance in the verification of the interleukin-2 gene as the basis of *Idd3* (Yamanouchi et al. 2007). Therefore, despite the difficulty in resolving the complex *Idd9.1/4* and *Idd9.2* loci, such genetic mapping studies remain of importance in defining disease susceptibility loci in both mouse and man.

## ^Electronic supplementary material^

^Below is the link to the electronic supplementary material.^

^Supplementary material 1 (PDF 135 kb)^

^Supplementary material 2 (XLSX 1776 kb)^

^Supplementary material 3 (PDF 218 kb)^

^Supplementary material 4 (PDF 185 kb)^
