# Supplementary material for: Fine mapping of type 1 diabetes regions Idd9.1 and Idd9.2 reveals genetic complexity
Source: Mamm Genome. 2013 Aug 11;24(9):358–75. doi: 10.1007/s00335-013-9466-y (PMC3824839; doi:10.1007/s00335-013-9466-y)
Supplement: Supplementary file 1 — Supplementary material 1 (PDF 135 kb) [file 335_2013_9466_MOESM1_ESM.pdf]

Supplemental table Ia: Markers that define the boundary of each line

Line Number	Proximal out marker	Proximal in marker	Distal in marker	Distal out marker
Line 1104	D4Mit27	D4Mit28	D4Mit42	rs32942538
Line 905	rs31864219	rs3676600	D4Mit42	rs32942538
Line 1565	rs31864219	rs3676600	D4Mit339	rs27543340
Line 2790 Proximal	rs31864219	rs3676600	D4Mit339	rs27543340
Line 2790 Distal	D4Mit127	AL606914(TGCA)	D4Mit42	rs32942538
Line 3571 Proximal	rs31864219	rs3676600	BqH15-T7	rs6361251
Line 3571 Distal	D4Mit127	AL606914(TGCA)	D4Mit42	rs32942538
Line 1106	D4Mit127	AL606914(TGCA)	D4Mit42	rs32942538
Line 928	rs6355453	rs13477984	D4Mit42	rs32942538
Line 1105	rs27604152	rs27628286	D4Mit42	rs32942538
Line 1566	CD30 distal 5	AL606963TA	Clstn1-RE	Pik3cd-RE
Line 9374	CD30 distal 5	AL606963TA	Chr4-147.563	Chr4-147.582
Line 6353	rs45830432	rs27615497	rs32076285	rs32566241
Line 930	rs45830432	rs27615497	rs13478063	rs32942538

Supplemental table Ib: Markers used for genotyping mice in this study

Marker	Position in Ensembl (GRCm38)	Forward	Reverse	Size	Comments
CD30 distal 5	145.378 Mb	ATGGAGAGAGTGAGGCAGGA	CTGTTCCCAGGTAGCCTTGA	200	microsatellite - agarose marker
AL606963TA	145.667 Mb	ATACCCAAAGCACCCTTGTG	GGTAGACTGCTCCAATGAAAGG	160	microsatellite - ABI3730 capillary sequencer
Chr4-147.563	148.189 Mb	GGGCTAGAATGGTCACTGGA	GCAGAGACTCAAAGGCCAAT	181	microsatellite - agarose marker
Chr4-147.582	148.208 Mb	CATCAACCCCAGGACAAAAG	AACGGGTTGATCCAGTCTGT	186	microsatellite - agarose marker
rs45830432	148.450 Mb	CCATGCTAGTACCCTGCACA	ACTCACAGGGCGTACTCGAT	169	RFLP NOD cuts with Hpych4IV
rs27615497	148.458 Mb	ACTGTCAGGCACCTTGTTCC	AGGCTGAACTGCAAACTGCT	159	RFLP cut with Alu1
rs32076285	148.769 Mb	ACAGCAGAGTGAGCTGGACA	CACCTCATTGCTGAGGGTTT	160	RFLP B10 cuts with Hpych4II
rs32566241	148.872 Mb	CTTTGAATCCCATCCCTTGA	GAAGCCCATGGAGAACTGAG	112	RFLP NOD cuts with BSR1
Clstn1-RE	149.581 Mb	GGGTAGCATCATCAGGTCACT	GGGCTCCAACAAACATTCTC	179	RFLP cut with HpyCH4IV
Pik3cd-RE	149.724 Mb	ACGCGCACTAATCCAACC	AGCTGCTCCTCTGTCCTGTG	107	RFLP cut with Aci1
AL606914(TGCA)	149.953 Mb	ATGAAGGCTTGGTGTCCTTG	AGAAAGCCAACTCCCTCCAG	205	microsatellite - ABI3730 capillary sequencer
BqH15-T7	128.688 Mb	AGCCTGTGTTACCCTGCAAT	CCCTCTCTCTTCCTGCACAA	185	microsatellite - ABI3730 capillary sequencer
rs13477984	131.623 Mb	CATGTGGTTCTGCTGCTGTC	CCTGGGGTGGCTGTGTATAA	152	SNP to sequence
rs13478063	153.325 Mb	GGAATGCCACTCCTCAGAGA	AACAGAACAGGGAGCCACAG	84	RFLP cut with HPYCH4III
rs27543340	134.937 Mb	CTGGGGTCTGAGAACCTGAA	TTCCCTGGATCACTGCTTTC	113	RFLP cut with HAEIII
rs27604152	141.456 Mb	CGACTCTGCACAGCATTGAT	CCTGAGGGAAGCACTGTAGC	98	RFLP cut with HpyAv
rs27628286	141.706 Mb	TTTCTCACCTGGGCTCACTC	ACCTTTCTTGCACGTTTTCC	114	RFLP cut with Taq1
rs31864219	116.875 Mb	GCAGGTGATAATCATCCCTTACTT	GCACAAGTACGCCTGTGTGT	107	SNP to sequence
rs32942538	154.341 Mb	TTTTGGTTTGGTGGTGAGGT	GTCAAGGCTGAGCCAAAGTC	90	RFLP cut with Msp1
rs3676600	119.767 Mb	AAACCAGGGTTCGTGCTATG	GTGACACCATGGCTCACAGA	347	SNP to sequence
rs6355453	131.080 Mb	GGACTCGAGGGACACACTTC	AGGAGTGGCTGGTCACAGAG	100	SNP to sequence
rs6361251	128.870 Mb	GAGGCAGATTTTGCACAGTTC	GGCCCACAATCTGCTTTTTA	207	SNP to sequence

Supplemental table Ic: Primers used for gene expression analysis

Gene	**Exons**	**Forward primer**	**Reverse primer**
Akr1e1	4-5	ACCCAGTCACACCAGCTTTC	CCTCAAACCAGGCTTATCCA
Akr1e1	8-9	TCCGATTTCAGATCCAAAGG	GGCTGAGGAGTTCCTCCATA
*Rex2-201**	1-3	CGGAGGAATGGGAATGTCT	GTGCTGTGTGTCTTGTTCCAA
*Rex2-202***	5-6	TTGCAGGACATTGTACATGGAT	GTGCTGTGTGTCTTGTTCCAA
Ccdc28b	2-3	ACTCCCTCATTTGCCATCAC	CTGTCACCTCTGTCAGGAAGG
Ptp4a2	2-4	AACTCACAACCCCACCAATG	AACGGCCAATCTAGAACGTG
Ptp4a2	3-4	CCAATGCGACTCTCAACAAG	AACGGCCAATCTAGAACGTG
Ptp4a2	4-6	TGTGTTGCAGTGCATTGTGT	AAAGCAGCTGTTTGGAATTGA
Rbbp4	3-4	TGTCCTGGGAACACACACAT	TGACAGAACCAAAACCTCCA
Rbbp4	6-7	CAGCAGTAGTGGAGGACGTG	AGTGGCTTGGCTTGGAAGTA
S100pbp	2-3	AGCGGGAGTTGAAACCTTTT	AGGCATTGCTTTTAGGCAGA
Zbtb8a	4-5	GGTTCAGCAGGCTAGACCAT	GGTTCCTTCCTGCACCACT
Zbtb8a	3-4	TCCCAGTCTGAAGAGCAGGT	ATGGGCATTTGAACCGAAG
β-actin	5-6	TGGAATCCTGTGGCATCCATGAAACC	TAAAACGCAGCTCAGTAACAGTCCG
β2m	2-4	AGACTGATACATACGCCTGCAG	GCAGGTTCAAATGAATCTTCAG

*Also primes Gm13138-001 and Gm13138-202

**Also primes Gm13138-201
