# Supplementary material for: Fine mapping of type 1 diabetes regions Idd9.1 and Idd9.2 reveals genetic complexity
Source: Mamm Genome. 2013 Aug 11;24(9):358–75. doi: 10.1007/s00335-013-9466-y (PMC3824839; doi:10.1007/s00335-013-9466-y)
Supplement: Supplementary file 3 — Supplementary material 3 (PDF 218 kb) [file 335_2013_9466_MOESM3_ESM.pdf]

Supplemental Table 3a. Consequences of SNP variations in protein-coding genes between NOD and C57BL/6 genomes in a portion of the *Idd9.1* region, 129.495-129.808 Mb.

Gene	Position m38	C57BL/6	NOD/ShiLtJ	AA change	Codon change	Consequence
Fam167b	129578336	T	C	K/E	Aag/Gag	Non synonymous coding
Dcdc2b	129608827	G	A	A/V	gCa/gTa	Non synonymous coding
Dcdc2b	129609105	G	T	R/S	Cgc/Agc	Non synonymous coding
Dcdc2b	129609645	T	TCCAGGGCTG	W/CSPG	tgg/tgCAGCCCTGGg	Codon change + 3 amino acid insertion
Dcdc2b	129609707	A	G	F/S	tTt/tCt	Non synonymous coding
Dcdc2b	129611279	C	T	A/T	Gcc/Acc	Non synonymous coding
Dcdc2b	129614068	C	T	A/T	Gct/Act	Non synonymous coding
Dcdc2b	129614109	A	G	V/A	gTc/gCc	Non synonymous coding
Iqcc	129616471	C	T	W/Stop	tGg/tAg	Stop gained resulting in 2aa truncation
Iqcc	129616487	G	A	R/C	Cgt/Tgt	Non synonymous coding
Iqcc	129616642	A	G	L/S	tTa/tCa	Non synonymous coding
Iqcc	129616648	T	C	N/S	aAt/aGt	Non synonymous coding
Iqcc	129616708	T	A	Q/L	cAa/cTa	Non synonymous coding
Iqcc	129616841	C	T	G/R	Gga/Aga	Non synonymous coding
Iqcc	129617753	T	C	D/G	gAt/gGt	Non synonymous coding
Iqcc	129617801	T	A	Q/L	cAg/cTg	Non synonymous coding
Ccdc28b	129622714	G	T	P/Q	cCa/cAa	Non synonymous coding
Tmem39b	129684417	T	C	H/R	cAc/cGc	Non synonymous coding

Supplemental Table 3b. Sequence variations in non-coding RNAs between NOD and C57BL/6 genomes in a portion of the *Idd9.1* region, 129.495-129.808 Mb.

Gene name	Position m38	C57BL/6	NOD/ShiLtJ	Description	Biotype
RP23-209C6.5	129509723	T	G	Novel Transcript, Antisense To A Novel Gene	antisense

Supplemental Table 3c. Consequences of SNP variations in protein-coding genes between NOD and C57BL/6 genomes in distal *Idd9.2* region.

Gene	Position [§]	C57BL/6	NOD/ShiLtj	AA change	Codon change	Consequence	Line 1566[§§]	Line 9374[§§]	Line 6353[§§]
Zfp933	147828455	G	T	A/D	gCc/gAc	Non synonymous coding			
Miip	147860963	T	A	Q/H	caA/caT	Non synonymous coding			
Miip	147860995	C	T	A/T	Gcc/Acc	Non synonymous coding			
Miip	147862304	T	C	Q/R	cAg/cGg	Non synonymous coding			
Miip	147865860	A	C	V/G	gTc/gGc	Non synonymous coding			
Miip	147865887	T	C	D/G	gAt/gGt	Non synonymous coding			
Miip	147865896	C	A	S/I	aGt/aTt	Non synonymous coding			
Fv1	147870050	G	A	E/K	Gaa/Aaa	Non synonymous coding			
Fv1	147870173	AG	GT	R/V	aga/GTa	Non synonymous coding			
Fv1	147870290	¶	¶¶	¶¶¶		Frame shift			
Mfn2	147878782	T	G	E/D	gaA/gaC	Non synonymous coding			
Mfn2	147885688	T	C	Q/R	cAg/cGg	Non synonymous coding			
Nppb	147986054	T	C	Y/H	Tat/Cat	Non synonymous coding			
Mthfr	148041660	A	G	S/G	Agc/Ggc	Non synonymous coding			
Agtrap	148080499	A	G	V/A	gTc/gCc	Non synonymous coding			
2610109H07Rik	148112777	T	C	T/A	Aca/Gca	Non synonymous coding			
2610109H07Rik	148115627	G	C	A/G	gCt/gGt	Non synonymous coding			
2610109H07Rik	148115747	G	C	T/S	aCt/aGt	Non synonymous coding			
Fbxo6	148149408	C	T	R/K	aGg/aAg	Non synonymous coding			
Fbxo44	148153519	G	A	R/W	Cgg/Tgg	Non synonymous coding			
Fbxo44	148153600	G	T	L/M	Ctg/Atg	Non synonymous coding			
Fbxo44	148156185	C	T	G/S	Ggt/Agt	Non synonymous coding			
Fbxo2	148164874	G	A	R/K	aGg/aAg	Non synonymous coding			
Ptchd2	148249782	G	A	S/L	tCg/tTg	Non synonymous coding			
Ptchd2	148255139	T	G	T/P	Act/Cct	Non synonymous coding			
Ptchd2	148271825	A	G	S/P	Tcc/Ccc	Non synonymous coding			
Ptchd2	148272323	T	C	I/V	Atc/Gtc	Non synonymous coding			
Ptchd2	148272346	G	T	P/Q	cCg/cAg	Non synonymous coding			

Ubiad1	148444348	G	A	L/F	Ctt/Ttt	Non synonymous coding			
Exosc10	148580410	A	G	I/V	Ata/Gta	Non synonymous coding			
Exosc10	148581780	G	T	C/F	tGt/tTt	Non synonymous coding			
Masp2	148603788	G	A	V/I	Gtt/Att	Non synonymous coding			
Masp2	148608001	TA	CG	I/T	ata/aCG	Non synonymous coding			
Masp2	148612097	G	C	E/Q	Gaa/Caa	Non synonymous coding			
Masp2	148614261	G	A	A/T	Gcc/Acc	Non synonymous coding			
Gm572	148668500	G	A	V/I	Gtt/Att	Non synonymous coding			
Gm572	148668600	T	C	M/T	aTg/aCg	Non synonymous coding			
Gm572	148668600	#	##	###		Frame shift			
Casz1	148929159	A	G	H/R	cAt/cGt	Non synonymous coding			
Casz1	148929467	A	G	T/A	Acc/Gcc	Non synonymous coding			
Casz1	148938614	A	G	M/V	Atg/Gtg	Non synonymous coding			
Casz1	148939231	T	G	I/M	atT/atG	Non synonymous coding			
Casz1	148939449	G	A	R/H	cGt/cAt	Non synonymous coding			
Casz1	148942940	G	A	S/N	aGc/aAc	Non synonymous coding			
Casz1	148944359	T	C	L/P	cTg/cCg	Non synonymous coding			
Casz1	148952306	*	-CAG	TA/T	acagcc/acc	Codon change/codon deletion			
Dffa	149106299	T	C	I/T	aTt/aCt	Non synonymous coding			
Cort	149125414	*	-CCACTCGTG	HEW/-	cacgagtgg/-	Codon deletion			
Apitd1	149128877	C	G	G/R	Gga/Cga	Non synonymous coding			
Pgd	149153903	C	G	G/A	gGc/gCc	Non synonymous coding			
Ube4b	149365481	T	A	E/D	gaA/gaT	Non synonymous coding			
Nmnat1	149468885	T	C	T/A	Acg/Gcg	Non synonymous coding			
Nmnat1	149469781	A	G	Y/H	Tac/Cac	Non synonymous coding			
Pik3cd	149698454	G	A			Splice site donor			

§ Position in GRCm38 build.
§§ Shaded area indicate extent of B10 derived congenic region in each strain.
¶ GGCTTGACTTCTGTAGGCTCTGTGGGGGTTCTCTCTCTCTCCCTGGAAACATCAAAGCAACAGTTAA
¶¶ ACAAAACTCTGA
¶¶¶ GLTSVGSVGVLSLSPWKHQSNS/TKL
# GGGGGGTGGGTCTCTCCAGCAGCCTGTGA
GGGTCTCTCCAGCAGCCTGTGAGCAGAGATAA
GVGLSSSL/VSPAACEQR

Supplemental Table 3d. Sequence variations in non-coding RNAs between NOD and C57BL/6 genomes in distal *Idd9.2* region.

Gene name	Position m38	C57BL/6	NOD/ShiLtJ	Description	Biotype
RP23-406N5.8	147807107	G	A	novel transcript	lincRNA
RP23-406N5.8	147807119	*	-T	novel transcript	lincRNA
RP23-406N5.8	147807123	C	A	novel transcript	lincRNA
RP23-406N5.8	147807126	T	A	novel transcript	lincRNA
RP23-406N5.8	147807130	A	G	novel transcript	lincRNA
RP23-406N5.8	147807132	G	T	novel transcript	lincRNA
RP23-406N5.8	147807166	T	G	novel transcript	lincRNA
RP23-406N5.8	147809668	C	G	novel transcript	lincRNA
RP23-406N5.8	147809703	G	C	novel transcript	lincRNA
RP23-406N5.8	147809714	C	A	novel transcript	lincRNA
RP23-406N5.8	147809748	G	A	novel transcript	lincRNA
RP23-406N5.8	147809751	*	-AAT	novel transcript	lincRNA
RP23-406N5.8	147809767	C	A	novel transcript	lincRNA
RP23-406N5.8	147809777	A	G	novel transcript	lincRNA
RP23-406N5.8	147809784	T	C	novel transcript	lincRNA
RP23-406N5.8	147809799	T	C	novel transcript	lincRNA
RP23-406N5.8	147809817	A	G	novel transcript	lincRNA
RP23-406N5.8	147809825	G	T	novel transcript	lincRNA
Gm13201	148071899	T	A	novel transcript, antisense to Mthfr (Gm13201)	antisense
Gm13201	148071907	A	G	novel transcript, antisense to Mthfr (Gm13201)	antisense
Gm13201	148073040	C	T	novel transcript, antisense to Mthfr (Gm13201)	antisense
Gm13206	148306941	G	A	novel transcript (Gm13206)	lincRNA
Gm13206	148315938	T	C	novel transcript (Gm13206)	lincRNA
Gm13206	148315959	C	T	novel transcript (Gm13206)	lincRNA
Gm13206	148316029	T	C	novel transcript (Gm13206)	lincRNA
Gm13206	148316034	G	A	novel transcript (Gm13206)	lincRNA
Gm13206	148316059	G	A	novel transcript (Gm13206)	lincRNA
Gm13206	148316127	*	+T	novel transcript (Gm13206)	lincRNA
Gm13206	148316229	A	G	novel transcript (Gm13206)	lincRNA
Gm13206	148316233	TC	GA	novel transcript (Gm13206)	lincRNA
Gm13206	148316272	T	C	novel transcript (Gm13206)	lincRNA
Gm13206	148316299	C	T	novel transcript (Gm13206)	lincRNA
Gm13206	148316310	A	T	novel transcript (Gm13206)	lincRNA
Gm13206	148316320	G	A	novel transcript (Gm13206)	lincRNA
Gm13206	148316355	G	A	novel transcript (Gm13206)	lincRNA

Gm13206	148316372	C	T	novel transcript (Gm13206)	lincRNA
Gm13206	148316448	A	G	novel transcript (Gm13206)	lincRNA
Gm13206	148316464	*	-GGGGTTACAG	novel transcript (Gm13206)	lincRNA
Gm13206	148316474	A	T	novel transcript (Gm13206)	lincRNA
Gm13206	148316509	*	-GGGCTTTCTCCTTAGCCCTAAAATTCAGACA AAACTATTAAACATAGATAAATTTTTTGCC	novel transcript (Gm13206)	lincRNA
Gm13200	148356411	C	T	novel transcript (Gm13200)	lincRNA
Gm13200	148356417	G	A	novel transcript (Gm13200)	lincRNA
Gm13209	148585014	C	A	novel transcript, antisense to Exosc10 (Gm13209)	antisense
Gm13209	148585862	G	C	novel transcript, antisense to Exosc10 (Gm13209)	antisense
Gm13209	148585872	*	-TTCT	novel transcript, antisense to Exosc10 (Gm13209)	antisense
Gm13209	148585999	A	G	novel transcript, antisense to Exosc10 (Gm13209)	antisense
Gm13203	148716277	T	C	novel transcript (Gm13203)	lincRNA
Gm13203	148716280	A	G	novel transcript (Gm13203)	lincRNA
Gm13203	148716285	T	C	novel transcript (Gm13203)	lincRNA
Gm13203	148716343	T	C	novel transcript (Gm13203)	lincRNA
Gm13203	148716391	A	T	novel transcript (Gm13203)	lincRNA
Gm13203	148716474	C	T	novel transcript (Gm13203)	lincRNA
Gm15969	148913566	T	C	novel transcript, antisense to Casz1 (Gm15969)	antisense
RP23-230I5.2	148947564	C	G	novel transcript, antisense to Casz1	antisense
RP23-230I5.2	148947601	C	T	novel transcript, antisense to Casz1	antisense
RP23-230I5.2	148947622	C	T	novel transcript, antisense to Casz1	antisense
RP23-230I5.2	148947641	G	A	novel transcript, antisense to Casz1	antisense
RP23-230I5.2	148947759	A	G	novel transcript, antisense to Casz1	antisense
RP23-230I5.2	148947832	G	A	novel transcript, antisense to Casz1	antisense
RP23-121D17.2	148961713	A	G	novel transcript, antisense to Pex14	antisense
RP23-121D17.2	148962237	*	+GGCTCAGA	novel transcript, antisense to Pex14	antisense
RP23-121D17.2	148962833	*	+A	novel transcript, antisense to Pex14	antisense
RP23-121D17.2	148962865	T	C	novel transcript, antisense to Pex14	antisense
RP23-121D17.2	148962885	T	C	novel transcript, antisense to Pex14	antisense
RP23-121D17.2	148962892	G	A	novel transcript, antisense to Pex14	antisense
RP23-121D17.2	148962976	A	G	novel transcript, antisense to Pex14	antisense
RP23-121D17.2	148962991	T	C	novel transcript, antisense to Pex14	antisense
RP23-121D17.2	148963215	C	T	novel transcript, antisense to Pex14	antisense
RP23-121D17.2	148963300	T	C	novel transcript, antisense to Pex14	antisense
RP23-121D17.2	148963551	G	T	novel transcript, antisense to Pex14	antisense
RP23-121D17.2	148966737	A	G	novel transcript, antisense to Pex14	antisense
RP23-121D17.2	148966809	CA	AT	novel transcript, antisense to Pex14	antisense

RP23-121D17.2	148972062	A	G	novel transcript, antisense to Pex14	antisense
RP23-121D17.2	148982095	A	G	novel transcript, antisense to Pex14	antisense
RP23-121D17.2	148982306	C	T	novel transcript, antisense to Pex14	antisense
RP23-121D17.2	148982449	A	G	novel transcript, antisense to Pex14	antisense
RP23-121D17.2	148982634	*	+ACACA	novel transcript, antisense to Pex14	antisense
RP23-121D17.2	148982759	*	+GAGCGAGCGAGCGAGCGAGAGAGA	novel transcript, antisense to Pex14	antisense
RP23-121D17.2	148982804	T	A	novel transcript, antisense to Pex14	antisense
RP23-121D17.2	148982913	A	G	novel transcript, antisense to Pex14	antisense
RP23-121D17.2	148983037	*	+T	novel transcript, antisense to Pex14	antisense
RP23-121D17.2	148983181	A	G	novel transcript, antisense to Pex14	antisense
RP23-121D17.2	148983393	A	G	novel transcript, antisense to Pex14	antisense
RP23-121D17.2	148983947	A	G	novel transcript, antisense to Pex14	antisense
RP23-173D8.3	149335721	C	T	novel transcript, antisense to Ube4b	antisense
RP23-173D8.3	149335817	A	T	novel transcript, antisense to Ube4b	antisense
Gm13069	149338142	C	T	novel transcript, antisense to Ube4b (Gm13069)	antisense
Gm13069	149338201	T	C	novel transcript, antisense to Ube4b (Gm13069)	antisense
Gm13069	149338219	*	-CC	novel transcript, antisense to Ube4b (Gm13069)	antisense
Gm13069	149338488	A	G	novel transcript, antisense to Ube4b (Gm13069)	antisense
Gm13069	149338496	A	G	novel transcript, antisense to Ube4b (Gm13069)	antisense
Gm13069	149338521	C	A	novel transcript, antisense to Ube4b (Gm13069)	antisense
Gm13069	149338530	*	+ATA	novel transcript, antisense to Ube4b (Gm13069)	antisense
RP23-173D8.2	149347912	T	C	novel transcript, antisense to Ube4b	antisense
RP23-173D8.2	149348036	G	A	novel transcript, antisense to Ube4b	antisense
RP23-173D8.2	149351970	*	-GCCACATCTCAGAGGGCCTCCT GCCAGAGGAGAGGCACAGAG	novel transcript, antisense to Ube4b	antisense
RP23-173D8.2	149352030	G	A	novel transcript, antisense to Ube4b	antisense
RP23-173D8.2	149352045	G	C	novel transcript, antisense to Ube4b	antisense
RP23-173D8.2	149352072	A	G	novel transcript, antisense to Ube4b	antisense
RP23-173D8.2	149352188	C	T	novel transcript, antisense to Ube4b	antisense
RP23-173D8.2	149352207	C	T	novel transcript, antisense to Ube4b	antisense
RP23-173D8.2	149357592	C	T	novel transcript, antisense to Ube4b	antisense
RP23-173D8.2	149357616	A	G	novel transcript, antisense to Ube4b	antisense
RP23-173D8.2	149358070	G	A	novel transcript, antisense to Ube4b	antisense
RP23-173D8.2	149358630	A	G	novel transcript, antisense to Ube4b	antisense
RP23-173D8.2	149358740	A	G	novel transcript, antisense to Ube4b	antisense
RP23-173D8.2	149358781	C	T	novel transcript, antisense to Ube4b	antisense
RP23-173D8.2	149358895	T	C	novel transcript, antisense to Ube4b	antisense
RP23-173D8.2	149359070	G	A	novel transcript, antisense to Ube4b	antisense

RP23-173D8.2	149359094	A	G	novel transcript, antisense to Ube4b	antisense
RP23-173D8.2	149359136	G	A	novel transcript, antisense to Ube4b	antisense
RP23-173D8.2	149359140	C	T	novel transcript, antisense to Ube4b	antisense
RP23-173D8.2	149359154	*	-A	novel transcript, antisense to Ube4b	antisense
RP23-173D8.2	149359272	A	G	novel transcript, antisense to Ube4b	antisense
RP23-173D8.2	149364842	T	A	novel transcript, antisense to Ube4b	antisense
RP23-173D8.2	149365157	T	C	novel transcript, antisense to Ube4b	antisense
Gm13097	149480459	G	A	novel transcript, antisense to Nmnat1 (Gm13097)	antisense
Gm13097	149482339	CC	TG	novel transcript, antisense to Nmnat1 (Gm13097)	antisense
Gm13097	149482378	G	A	novel transcript, antisense to Nmnat1 (Gm13097)	antisense
Gm13097	149483315	A	G	novel transcript, antisense to Nmnat1 (Gm13097)	antisense
Gm13097	149483446	*	+CCT	novel transcript, antisense to Nmnat1 (Gm13097)	antisense
Gm13097	149483479	C	T	novel transcript, antisense to Nmnat1 (Gm13097)	antisense
Gm13097	149483497	A	G	novel transcript, antisense to Nmnat1 (Gm13097)	antisense
Gm13066	149565709	T	C	novel transcript, antisense to Ctnnbip1 (Gm13066)	antisense
Gm13068	149749429	*	+CTT	novel transcript, antisense to Slc25a33 (Gm13068)	antisense
Gm13068	149749583	C	T	novel transcript, antisense to Slc25a33 (Gm13068)	antisense
Gm13073	149780071	A	G	novel transcript (Gm13073)	lincrna
Gm13073	149780119	T	C	novel transcript (Gm13073)	lincrna
Gm13073	149780120	*	-GG	novel transcript (Gm13073)	lincrna
Gm13073	149780164	*	-G	novel transcript (Gm13073)	lincrna
Gm13073	149780597	T	C	novel transcript (Gm13073)	lincrna
