# Supplementary material for: Fine mapping of type 1 diabetes regions Idd9.1 and Idd9.2 reveals genetic complexity
Source: Mamm Genome. 2013 Aug 11;24(9):358–75. doi: 10.1007/s00335-013-9466-y (PMC3824839; doi:10.1007/s00335-013-9466-y)
Supplement: Supplementary file 4 — Supplementary material 4 (PDF 185 kb) [file 335_2013_9466_MOESM4_ESM.pdf]

Supplemental Table 4a. Differentially expressed probes from the Affymetrix core probe set.

Probe ID	Gene Symbol	Chromosome	Start position	*Idd9* region	Fold change	Magnitude fold change	Adjusted P Value	Average Expression	Mean NOD	Mean Idd9
6810717	Akr1e1	13	4590767		4.05	4.05	1.50E-06	166.7450357	6.372666667	8.390333333
6918533	Rex2	4	146964351	9.2	0.45	2.24	0.00185219	97.59035504	7.192	6.025333333
6923411	Jun	4	95049053		0.49	2.05	0.003658532	271.7871304	8.602666667	7.57
6980101	Cd209d	8	3871825		1.77	1.77	0.005262629	117.0109715	6.458333333	7.282666667
6940358	Plac8	5	100553733		1.82	1.82	0.006733638	256.0591554	7.568	8.432666667
6899428	Sprr2d	3	92339140		0.57	1.75	0.006801842	196.6745765	8.021333333	7.218
6904300	Ccna2	3	36564872		1.75	1.75	0.006801842	373.9841541	8.142333333	8.951333333
6925587	Ccdc28b	4	129619274	9.1	1.91	1.91	0.006801842	736.8191087	9.057333333	9.993
6791298	Top2a	11	98937383		1.92	1.92	0.006801842	349.5447444	7.978666667	8.92
6998900	Tdgf1	9	110931759		2.09	2.09	0.008524053	165.7655039	6.84	7.906
6815558	Ccnb1	13	100778670		1.76	1.76	0.011304529	208.8755765	7.300666667	8.112333333
6957304	Prmt8	6	127689011		0.52	1.92	0.011771418	35.79865137	5.633666667	4.69
6913499	Smc2	4	52369442		1.62	1.62	0.012689761	282.9035885	7.798333333	8.49
6772417	Tnfaip3	10	18860936		0.55	1.82	0.016027113	243.2562957	8.358	7.494666667
6786044	Fignl1	11	11776658		1.54	1.54	0.016027113	268.9138033	7.759666667	8.382333333
6790475	Brip1	11	86058136		1.61	1.61	0.016027113	109.0238528	6.425333333	7.111666667
6791978	Kpna2	11	106988649		1.67	1.67	0.016027113	206.4525842	7.318333333	8.061
6758663	Asnsd1	1	53325517		1.65	1.65	0.016985449	243.0596607	7.566	8.284333333
6879938	Ccdc34	2	110017837		1.69	1.69	0.016985449	178.6922597	7.101666667	7.861
6910611	Dnajb4	3	152165501		0.58	1.72	0.020581596	365.7379866	8.905666667	8.123666667
6926082	Rcan3	4	135412312	9.1	0.59	1.7	0.020581596	179.4369674	7.868	7.106666667
6757634	Ptp4a1	1	30940306		0.64	1.57	0.020581596	507.4657577	9.313666667	8.660666667
7017550	Xlr3a	X	73086296		1.59	1.59	0.020581596	109.4402788	6.439666667	7.108333333
6811530	Hist1h1b	13	21779887		1.6	1.6	0.020581596	109.7314553	6.438	7.117666667
6780933	Vdac1	11	52332877		1.61	1.61	0.020581596	318.1727932	7.968333333	8.659
7018897	Nsbp1	X	109004430		1.61	1.61	0.020581596	333.2589937	8.038666667	8.722333333
6880497	Rad51	2	119112627		1.65	1.65	0.020581596	137.1870032	6.74	7.46
6857078	Ndc80	17	71496105		1.57	1.57	0.020632235	190.9211781	7.251	7.902666667
6919748	Fam92a	4	12100669	9.1	1.61	1.61	0.022478566	296.2832656	7.868333333	8.553333333
6897002	Plk4	3	40800038		1.52	1.52	0.02251174	206.5480076	7.386	7.994666667
6959568	Polr2i	7	30231779		1.56	1.56	0.02251174	172.4458978	7.11	7.75
6960178	Klk8	7	43797413		1.6	1.6	0.02251174	294.7470029	7.864333333	8.542333333

6895672	Fabp5	3	9932449		1.59	1.59	0.02498998	252.5922678	7.645333333	8.316
6936981	Tyms	5	30044475		1.62	1.62	0.02498998	215.3192259	7.401	8.099666667
6807022	Cks2	13	51600235		1.65	1.65	0.02498998	358.915396	8.127666667	8.847333333
6925559	Zbtb8a	4	129353633	9.1	0.64	1.56	0.025648563	92.95754096	6.858333333	6.218666667
6989974	2810417H13Rik	9	65890333		1.58	1.58	0.025648563	282.7728895	7.813666667	8.473333333
6971227	Nsmce1	7	125467646		1.51	1.51	0.026596871	373.2072839	8.246333333	8.841333333
6936082	Dbf4	5	8396899		1.66	1.66	0.026596871	167.4592974	7.023333333	7.752
6980158	Shcbp1	8	4735986		1.82	1.82	0.026612717	127.8079115	6.566666667	7.429
6819629	Kcnrg	14	61607457		1.98	1.98	0.026612717	256.6811129	7.511	8.496666667
6929813		5	33653358		1.49	1.49	0.02719704	197.243416	7.336666667	7.911
6981683	Thex1	8	35405141		1.53	1.53	0.029258196	413.2392081	8.385666667	8.996
6805273	Hist1h2bj	13	22043230		1.71	1.71	0.029469826	440.907844	8.398666667	9.17
6971848	Mki67	7	135689791		1.64	1.64	0.030835518	737.6708074	9.169666667	9.884
6854487	Dusp1	17	26505604		0.55	1.81	0.030893942	451.0051196	9.246	8.388
6946778	Mad2l1	6	66535317		1.56	1.56	0.032509875	372.7763869	8.221333333	8.863
6994624	Hyls1	9	35560820		1.57	1.57	0.033036547	178.3416673	7.155	7.802
6832153	Xrcc6	15	81987857		1.46	1.46	0.033213681	198.5924088	7.362	7.905333333
6937254	Slbp	5	33630473		1.45	1.45	0.03502091	321.7210819	8.063	8.596333333
6869577	Hells	19	38930944		1.61	1.61	0.035087935	241.2134706	7.57	8.258333333
6976320	Hmgb2	8	57511596		1.68	1.68	0.035454566	702.9768322	9.081	9.833666667
6950125	Clec2d	6	129112941		1.71	1.71	0.037698183	671.0764564	9.001333333	9.779333333
6999549	Higd1a	9	121848563		1.55	1.55	0.039931951	414.5301806	8.377666667	9.013
6925254	Cdca8	4	124917678	9.1	1.77	1.77	0.039931951	193.8548921	7.185	8.012666667
6798795	Rhob	12	8497673		0.68	1.47	0.041397763	134.7834921	7.351	6.798
6977151	Klf2	8	72319043		0.71	1.41	0.041397763	391.4904578	8.861333333	8.364333333
6835973	Tmem65	15	58820402		1.41	1.41	0.041397763	152.9412047	7.008333333	7.505333333
6844362	Mrpl40	16	18872037		1.42	1.42	0.041397763	250.0662705	7.711	8.221333333
6887520	Spc25	2	69193907		1.44	1.44	0.041397763	161.6989686	7.072666667	7.601666667
6830165	Ebag9	15	44619164		1.45	1.45	0.041397763	299.9683892	7.958333333	8.499
6878053	Cdca7	2	72476203		1.46	1.46	0.041397763	203.0230892	7.394666667	7.936333333
6755378	Kmo	1	175620401		1.5	1.5	0.041397763	133.7288257	6.772	7.354333333
6927456	Cnga1	5	72603716		1.51	1.51	0.041397763	160.6191389	7.029333333	7.625666667
6869503	Kif11	19	37376124		1.54	1.54	0.041397763	156.5883784	6.981666667	7.6
6880468	D2Ertd750e	2	118813988		1.56	1.56	0.041397763	168.3904682	7.075666667	7.715666667
6960834	Siglech	7	55732854		1.65	1.65	0.041397763	172.226898	7.068333333	7.788
6815870	Ndufaf2	13	108002725		1.45	1.45	0.042439102	476.8857951	8.627333333	9.167666667

6922528	Stmn1	4	69925210		1.43	1.43	0.042851694	880.7975628	9.527	10.03833333
6805245	Hist1h2bn	13	21754123		1.47	1.47	0.042851694	453.5653647	8.545	9.105333333
6901592	Cenpe	3	135212557		1.51	1.51	0.043934082	140.7346079	6.839666667	7.434
6941344	Cit	5	116006776		1.46	1.46	0.044080768	241.2134706	7.639666667	8.188666667
6811762	Gmnn	13	24751848		1.45	1.45	0.044479327	258.6456887	7.749	8.280666667
6953443	Igf2bp3	6	49085223		1.41	1.41	0.045705669	118.425285	6.639333333	7.136333333
6894180	Cables2	2	180258549		1.46	1.46	0.045705669	143.3435653	6.89	7.436666667
6917045	Inpp5b	4	124741870	9.1	1.51	1.51	0.045705669	395.1253689	8.328333333	8.924
6873282	Ndufb8	19	44548582		1.53	1.53	0.048657011	455.0874528	8.525333333	9.134666667
6957731	E330021D16Rik	6	136400315		1.4	1.4	0.04978776	53.62498126	5.501	5.988666667
6861880	Stard6	18	70472474		1.4	1.4	0.04978776	92.05983808	6.282	6.767
6970635	Rras2	7	114044676		1.48	1.48	0.04978776	275.3901853	7.820666667	8.39

Supplemental Table 4b: Differentially expressed probes from the Affymetrix extended probe set.

Probe ID	Gene Symbol	Chromosome/Probe location	*Idd9* region	Fold change	Magnitude Fold Change	Adjusted P value	Average Expression	Mean NOD	Mean Idd9
6918433		chr4:144423416-144423701		0.122116778	8.188882951	1.05E-08	238.9944546	9.418	6.384
6867232		chr18:85140066-85140672		0.173860246	5.751746131	1.37E-08	181.6477896	8.767	6.243
6926806	1700029I01Rik	chr4:147625873-147676053	9.2	0.154534408	6.471050787	1.37E-08	151.4467351	8.589	6.080
6808990		chr13:93198975-93199718		5.718618511	5.718618511	5.42E-07	132.28448	5.789	8.305
7007305		chr4:145796614-145796815	9.2	3.951316288	3.951316288	5.48E-07	295.2582034	7.215	9.197
6810717	Akr1e1	chr13:4590767-4609179		3.73126976	3.73126976	1.35E-06	157.7139722	6.351	8.251
7008719	OTTMUSG00000011027	chr4:145538644-145539242	9.2	0.283024726	3.533260206	2.02E-06	95.88057572	7.493	5.672
6918564		chr4:147167701-147168321	9.2	3.248258992	3.248258992	8.20E-06	273.0144182	7.243	8.943
6918571		chr4:147186533-147187245	9.2	3.750284386	3.750284386	1.80E-05	194.6627908	6.651	8.558
6758507		chr1:48731997-48796216		4.007400407	4.007400407	2.25E-05	139.8109293	6.126	8.129
6918458		chr4:145817102-145817724	9.2	2.928171392	2.928171392	4.41E-05	278.4613589	7.346	8.896
7001443		chr13:68436661-68437226		0.356506429	2.804998501	4.41E-05	159.4175517	8.060	6.572
6867440		chr18:6147086-6147327		5.258555916	5.258555916	4.81E-05	139.7463379	5.929	8.324
6895778	OTTMUSG00000010173	chr3:12744194-12873679		0.363073448	2.754263649	5.86E-05	129.0840257	7.743	6.281
6925576		chr4:129563018-129563645	9.1	3.400310911	3.400310911	5.86E-05	422.6051876	7.840	9.606
6926693	1700095A21Rik	chr4:145681984-145711927	9.2	0.368652471	2.712581843	5.97E-05	97.96310976	7.334	5.894
7020895	G530011O06Rik	chrX:169974421-169975877		0.37310838	2.680186388	7.54E-05	227.9910425	8.544	7.122
6777345		chr10:117683535-117684293		2.964249054	2.964249054	9.16E-05	192.2491399	6.803	8.371
6910291	Rpl11	chr3:146838811-146844392		0.374663325	2.669062953	9.51E-05	1629.07051	11.378	9.961
6918408	OTTMUSG00000011027	chr4:145538644-145539242	9.2	0.342854097	2.916692579	9.51E-05	89.58384393	7.257	5.713
7008721	OTTMUSG00000010173	chr19:9273804-9273855	9.2	0.342616531	2.918714977	9.51E-05	120.1200636	7.681	6.136
6973711		chr8:3871834-3872208		2.531513188	2.531513188	9.51E-05	116.2429975	6.191	7.531
7017793		chrX:78047563-78048459		0.388413881	2.574573279	9.51E-05	135.0016597	7.759	6.395
6918463		chr4:145831995-145832501	9.2	2.936301272	2.936301272	0.000118855	127.2039799	6.214	7.768
6847427		chr16:82207684-82210722		2.624422509	2.624422509	0.000118855	272.9828802	7.397	8.789
6918537		chr4:146976318-146976901	9.2	0.353880294	2.825814311	0.000174575	121.1513497	7.67	6.171
6918568		chr4:146976318-146976901	9.2	2.511704368	2.511704368	0.000174575	128.3553838	6.340	7.668
6918447		chr4:145743883-145744434	9.2	0.397033689	2.518677954	0.000198463	82.46252126	5.699	6.618
6926070		chr4:135180700-135181264		0.375964062	2.659828696	0.000198463	188.2274638	8.262	6.851
6799279		chr12:17547157-17547735		0.405376211	2.466844312	0.000212928	347.1302625	9.091	7.788
6808991		chr13:93201706-93202701		2.982798801	2.982798801	0.00021527	180.643308	6.709	8.285
6926836		chr4:147755991-147756235	9.2	0.360982299	2.770218936	0.000265943	704.765748	10.196	8.726
6981791		chr8:37384292-37390042		2.726405215	2.726405215	0.000276561	247.8230669	8.677	7.938

6918436		chr4:145687617-145688045	9.2	0.423372656	2.361985323	0.000367484	77.15387258	5.650	6.939
6917875		chr4:137014517-137015129		2.323554257	2.323554257	0.000481516	199.7197691	7.033	8.25
6855140		chr17:35870814-35871298		2.30910429	2.30910429	0.00096833	182.4469639	6.908	8.115
6771025		chr10:117657170-117657495		2.225814454	2.225814454	0.001086578	93.10800687	7.118	5.964
6918533	Rex2	chr4:146964351-147423406	9.2	0.426022048	2.347296357	0.001253138	119.0562873	6.28	7.511
6918519		chr4:146164014-146164514	9.2	0.450417046	2.220164641	0.001284496	116.7274452	7.442	6.292
6749408		chr1:52177473-52178087		0.469218998	2.131201003	0.001592389	255.3796929	8.542	7.451
7000988	Mid1	chr16:36364212-36364313		0.247929183	4.03340982	0.001856703	85.52850001	7.424	5.412
6918455		chr4:145794038-145794531	9.2	2.570412675	2.570412675	0.001856921	130.6291746	6.348	7.710
6771024	Cpm	chr10:117655572-117687352		2.133664486	2.133664486	0.001885809	125.51071	6.425	7.518
6754684		chr1:164083959-164084166		0.424155939	2.357623479	0.001885809	99.2962044	7.252	6.015
6749585		chr1:55523081-55523236		0.410750243	2.434569467	0.001998059	73.93403091	6.85	5.566
6802804		chr12:93541831-93542000		2.229417273	2.229417273	0.002364338	116.7813972	6.289	7.446
6760745		chr1:92925334-92925735		0.486439852	2.055752619	0.003585444	250.4131764	8.488	7.448
6918644		chr4:147480240-147480736	9.2	0.463294031	2.158456473	0.003971405	229.9750168	8.400	7.290
6926027		chr4:134741574-134742232	9.1	0.469869925	2.128248578	0.003971405	105.5049346	7.266	6.176
6923411	Jun	chr4:95049053-95055499		0.490162946	2.040137892	0.004222109	272.2270592	8.603	7.574
6871550		chr19:11448114-11448659		2.278366754	2.278366754	0.004600774	554.3551253	8.521	9.709
